# Suicide, self-harm and thoughts of suicide or self-harm in infectious disease epidemics: a systematic review and meta-analysis

**DOI:** 10.1017/S2045796021000214

**Published:** 2021-04-27

**Authors:** J. P. Rogers, E. Chesney, D. Oliver, N. Begum, A. Saini, S. Wang, P. McGuire, P. Fusar-Poli, G. Lewis, A. S. David

**Affiliations:** 1 Division of Psychiatry, University College London, London, UK; 2 South London and Maudsley NHS Foundation Trust, London, UK; 3 Department of Psychosis Studies, King's College London, London, UK; 4 GKT School of Medical Education, King's College London, London, UK; 5 Medical School, University College London, London, UK; 6 Department of Psychology, King's College London, London, UK; 7 Department of Brain and Behavioral Sciences, University of Pavia, Pavia, Italy; 8 UCL Institute of Mental Health, University College London, London, UK

**Keywords:** systematic review, suicide, self-harm, infection, epidemic

## Abstract

**Aims:**

Suicide accounts for 2.2% of all years of life lost worldwide. We aimed to establish whether infectious epidemics are associated with any changes in the incidence of suicide or the period prevalence of self-harm, or thoughts of suicide or self-harm, with a secondary objective of establishing the frequency of these outcomes.

**Methods:**

In this systematic review and meta-analysis, MEDLINE, Embase, PsycINFO and AMED were searched from inception to 9 September 2020. Studies of infectious epidemics reporting outcomes of (a) death by suicide, (b) self-harm or (c) thoughts of suicide or self-harm were identified. A random-effects model meta-analysis for the period prevalence of thoughts of suicide or self-harm was conducted.

**Results:**

In total, 1354 studies were screened with 57 meeting eligibility criteria, of which 7 described death by suicide, 9 by self-harm, and 45 thoughts of suicide or self-harm. The observation period ranged from 1910 to 2020 and included epidemics of Spanish Flu, severe acute respiratory syndrome, human monkeypox, Ebola virus disease and coronavirus disease 2019 (COVID-19). Regarding death by suicide, data with a clear longitudinal comparison group were available for only two epidemics: SARS in Hong Kong, finding an increase in suicides among the elderly, and COVID-19 in Japan, finding no change in suicides among children and adolescents. In terms of self-harm, five studies examined emergency department attendances in epidemic and non-epidemic periods, of which four found no difference and one showed a reduction during the epidemic. In studies of thoughts of suicide or self-harm, one large survey showed a substantial increase in period prevalence compared to non-epidemic periods, but smaller studies showed no difference. As a secondary objective, a meta-analysis of thoughts of suicide and self-harm found that the pooled prevalence was 8.0% overall (95% confidence interval (CI) 5.2–12.0%; 14 820 of 99 238 cases in 24 studies) over a time period of between seven days and six months. The quality assessment found 42 studies were of high quality, nine of moderate quality and six of high quality.

**Conclusions:**

There is little robust evidence on the association of infectious epidemics with suicide, self-harm and thoughts of suicide or self-harm. There was an increase in suicides among the elderly in Hong Kong during SARS and no change in suicides among young people in Japan during COVID-19, but it is unclear how far these findings may be generalised. The development of up-to-date self-harm and suicide statistics to monitor the effect of the current pandemic is an urgent priority.

## Introduction

Suicide, the intentional ending of a person's own life, accounts for approximately 817 000 deaths and 2.2% of all years of life lost worldwide annually (Naghavi and Global Burden of Disease Self-Harm Collaborators, 2019). Self-harm is a broader concept, which encompasses degrees of intentionality that are hard to separate: from attempted suicide (which the World Health Organization (WHO) estimates dwarfs death by suicide by at least 20-fold) to non-suicidal self-injury (Gvion and Apter, 2012; World Health Organization, 2014). Thoughts of suicide is also a complex area and these are sometimes considered in terms of active thoughts of suicide (considering intentionally ending one's life) and passive thoughts of suicide (thoughts about not wishing to live any longer) (Beck *et al*., 1979). The epidemiology of suicide and self-harm show marked differences in terms of age, gender and culture (Skegg, 2005; Colucci and Martin, 2007; Angst *et al*., 2014; Fazel and Runeson, 2020).

An epidemic occurs when a disease significantly exceeds the expected number of cases in a given population. A pandemic is an epidemic that occurs over multiple countries or continents (U.S. Department of Health and Human Services, 2011). Infectious epidemics can be caused by a large range of pathogens, including viruses, bacteria, parasites and prions (‘WHO | Disease Outbreaks by Year’ (WHO, 2020)). The current COVID-19 pandemic is caused by the SARS-CoV-2 virus and has spread with unprecedented speed, resulting in intense speculation as to its effects on physical and mental health (Douglas *et al*., 2020; Holmes *et al*., 2020; Rogers *et al*., 2020; Wang and Tang, 2020).

Disasters and existential threats may result in higher rates of suicide and there was some evidence that suicide rates increased during the 2008 financial crisis in Europe (Parmar *et al*., 2016). However, this is not automatic and rates may even fall, perhaps due to increased social cohesion (Lester, 1994; Claassen *et al*., 2010), as postulated by Durkheim in the 19th century (Durkheim, 1897). There is a reason for concern about the impact of infectious outbreaks on the frequency of suicide and self-harm. Possible reasons for an increase include fear of infection, the stigma for those infected, pressure on health care systems – with a detrimental impact on health care workers – financial pressures, unemployment, social isolation, increased stress on intimate relationships, increasing access to lethal means, worsening substance misuse and media alarmism (Aquila *et al*., 2020*a*, 2020*b*; Fusar-Poli *et al*., 2020; Kawohl and Nordt, 2020; Moutier, 2020; Reger *et al*., 2020; Salazar de Pablo *et al*., 2020; Wasserman *et al*., 2020). Concerns have also been raised about the particular impact on certain groups, namely those actually infected (Rogers *et al*., 2020), health care workers (Aquila *et al*., 2020*a*; Reger *et al*., 2020; Salazar de Pablo *et al*., 2020), those with pre-existing psychiatric illness (Gunnell *et al*., 2020) and the elderly (Aquila *et al*., 2020*a*). Case series of suicides that are apparently related to the COVID-19 pandemic have emerged from India, Germany and Pakistan, highlighting issues of pre-existing mental health problems, fear of the pandemic, financial and occupational problems, loneliness, stigma related to infection and alcohol withdrawal (Ahmed *et al*., 2020; Buschmann and Tsokos, 2020; Dsouza *et al*., 2020; Mamun and Ullah, 2020; Rajkumar, 2020; Shoib *et al*., 2020; Syed and Griffiths, 2020).

The International Association for Suicide Prevention has noted the paucity of data on the effects of the current pandemic on suicide and has issued an urgent call for further evidence on the subject (IASP Executive Committee, 2020). One systematic review examined the psychological experience of survivors of Ebola virus disease and reported a high frequency of thoughts of suicide in a small population (Keita *et al*., 2017; James *et al*., 2019). A recently published review examined suicide in viral epidemics, but articles published after 7 April 2020 were excluded, so it does not consider evidence from the COVID-19 pandemic (Leaune *et al*., 2020). Another review including articles up to May 2020 was also unable to include any peer-reviewed studies on COVID-19 and did not conduct any meta-analysis (Zortea *et al*., 2020). To our knowledge, this is the most comprehensive review of suicide and self-harm in infectious epidemics and the first to include substantial data on the COVID-19 pandemic.

This review aimed to assess the impact of infectious epidemics on individuals in the geographical area of the epidemic (whether or not they were infected) in terms of actual cases of suicide, self-harm, and thoughts of suicide or self-harm, both during the epidemic and in the subsequent two-year period, during which time the effects may still be felt economically and socially. We also aimed to identify any risk factors that would highlight especially vulnerable groups.

## Method

### Objectives

We aimed to establish whether – during an infectious epidemic – there is a change in rates of (a) death by suicide, (b) self-harm, and (c) thoughts of suicide or self-harm. The population was people in a region where an infectious epidemic took place. The comparison groups (where available) were either the same population during a different time period or a different population during the same time period. Our primary outcome was the change in death by suicide; secondary outcomes were self-harm and thoughts of suicide or self-harm. An additional objective was to establish the frequency of these three outcomes during an infectious epidemic. We initially intended to meta-analyse the incidences of these outcomes, but, while incidences for death by suicide were available, the other outcomes were generally reported in terms of period prevalences. In such cases, meta-analysis of period prevalence was completed instead. Studies were included if they reported original research published in peer-reviewed journals and they described randomised controlled trials, cohort studies, case-control studies, cross-sectional surveys or ecological studies.

We included studies that reported suicide, suicide attempts, non-suicidal self-injury, thoughts of suicide and thoughts of self-harm, either self-reported or elicited by a clinician. However, many studies did not distinguish between these outcomes. Specifically, suicide attempts and non-suicidal self-injury were not always distinguished, and thoughts of suicide and thoughts of self-harm were not always distinguished (often because studies used a measure, such as the Patient Health Questionnaire-9 (PHQ-9) that includes both symptoms in a single question). We, therefore, reported our outcomes in three groups: (a) death by suicide, (b) self-harm (including suicide attempts and non-suicidal self-injury) and (c) thoughts of suicide or self-harm. We also included studies that examined internet search trends for suicide-related terms, as a proxy measure for thoughts of suicide.

### Search strategy and selection criteria

This systematic review followed Preferred Reporting Items for Systematic Reviews and Meta-Analyses (PRISMA) guidelines, as shown in the online Supplementary material (pp. 2–5). The study review protocol was pre-registered on the PROSPERO database and is available at https://www.crd.york.ac.uk/prospero/display_record.php?ID=CRD42020193926.

We included original studies that reported suicide, suicide attempts, actual self-harm, thoughts of suicide, or thoughts of self-harm among populations where an infectious epidemic had occurred, either during or in the two years following the epidemic. We examined for control groups that were either the same population during a different time period or a different population during the same time period. We did, however, include studies that lacked control groups, as they have value in calculating pooled prevalences. There were no exclusions based on language; where a paper was not in English, screening and data extraction were conducted in consultation with a co-author who was a native speaker of that language. In order to enable us to observe the relationship between exposure and outcomes, we made a pragmatic decision to exclude epidemics (such as HIV) that lasted longer than three years and events recorded more than two years after the end of an epidemic.

We used OVID to search MEDLINE (and Epub ahead of print, in-process and other non-indexed citations, Daily and Versions(R)), Embase (Classic + Embase), APA PsycINFO and AMED (Allied and Complementary Medicine) from inception until 24 June 2020; the search was subsequently updated to 9 September 2020. The overall search strategy was to combine epidemic AND infection AND (suicide OR self-harm), along with the comprehensive use of synonyms and subject headings to search within titles, abstracts and keywords without limits. The entire search strategy is in the online Supplementary material (pp. 9–16). In addition, we searched the reference lists of other relevant papers, examined the references from a related live systematic review (https://f1000research.com/articles/9-1097) and contacted experts in the field to identify unpublished data.

De-duplication was conducted manually by one reviewer (NB) in consultation with a second (JPR). Two reviewers (JPR and EC) screened titles, abstracts and full texts of extracted articles sequentially. Where there was disagreement on the inclusion of a title or abstract, it was retained for the next round of screening. Where there was disagreement on the inclusion of a full text, it was discussed with a third reviewer (DO). Reasons for exclusion of full texts were recorded.

### Data extraction

Data extraction included the citation, geographical region, infection, date of the epidemic, study design, data collection method, population, control group, number of cases, number of controls, age, gender, time period, outcomes reported, and number of individuals with each outcome. Data for each paper were independently extracted by two of the authors (EC, NB, AS and JPR). Where reviewers disagreed, a third author (JPR or EC) arbitrated. Where there were missing data, investigators were contacted with a request to provide these data.

### Data analysis

A systematic review of the literature was conducted, summarising results with one table for each of the three specified outcomes. The meta-analysis was also planned for each of these outcomes if at least five studies with relevant data were available. Studies were included in meta-analyses where there was the systematic assessment of outcomes for every individual. A random-effects model was employed because high heterogeneity was expected. A logit transformation was used to better approximate a normal distribution, as required by the assumptions of conventional meta-analysis models. Following the analysis, the synthesised result was back-transformed for ease of interpretation. The effect size measure for death by suicide was incidence; the effect size measure for self-harm or thoughts of suicide or self-harm was period prevalence. Period prevalences were defined as the proportion of cases over the sample size during the stated period (Barendregt *et al*., 2013). If data from multiple populations (e.g. patients and healthy controls) were reported, these were considered as separate estimates of period prevalence in the analysis. Due to a lack of studies presenting data from a control group, we were unable to assess change in incidence or prevalence in the meta-analysis. Subgroup analysis was planned by geographical region, specific disease epidemic, age group, gender, outcome operationalisation (thoughts of self-harm *v*. thoughts of suicide) and presence of pre-existing mental disorder. Actual subgroup analysis was performed for age group (children and adolescents), pre-existing mental disorder, infection status, health care worker status and phase of the epidemic (intra-epidemic *v*. post-epidemic) with a meta-regression for outcome operationalisation. *I*^2^ was calculated as a measure of between-study heterogeneity. Funnel plot asymmetry was not assessed as meta-analysis was used to calculate pooled prevalence, which is not characterised by the potential for negative or undesirable results that could have biased publication (Sterne *et al*., 2011). To assess the robustness of the results, we performed sensitivity analyses by sequentially removing each study and re-running the analysis. We also performed a sensitivity analysis by study quality. Meta-regressions to investigate the impact of the type of assessment (thoughts of suicide plus thoughts of self-harm *v*. thoughts of suicide alone) were conducted on a subgroup level if more than ten studies reported relevant data on the same outcome. Data were analysed using *R* version 3.3.2 and the meta-package version 4.11. The threshold for two-tailed statistical significance was set to *p* < 0.05.

Assessment of quality and risk of bias were performed at the study level using the Newcastle-Ottawa Scale (Wells *et al*., 2000). Studies scoring 0–3 point were deemed to be of low quality, those scoring 4–6 were of moderate quality and those scoring 7–9 were of high quality.

## Results

**Fig. 1.**
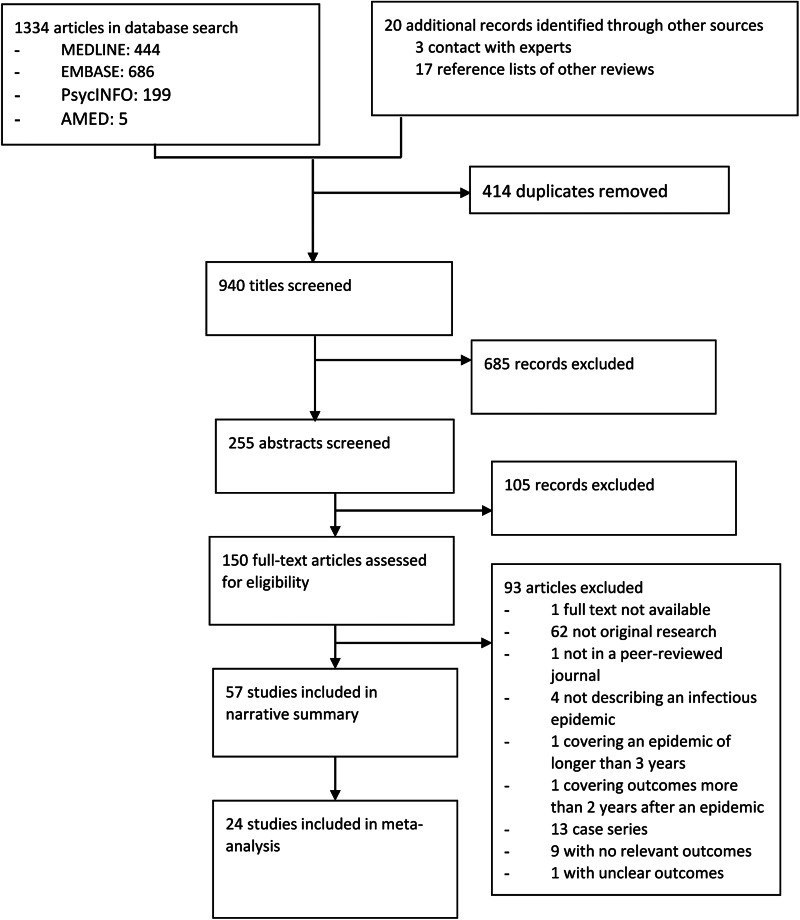
PRISMA diagram.

In total, 1354 articles were screened with 57 meeting eligibility criteria, as shown in Fig. 1, 32 of which were added after the literature search was re-run 77 days later. Seven studies described suicide, 9 self-harm and 45 thoughts of suicide or self-harm (some studies reporting more than one relevant outcome). Sample size ranged between 21 and ~87 000 000. The mean age of the samples, where reported, ranged from 19.9 years [standard deviation (s.d.) 1.6] to 74.9 years (s.d., 5.7). The date of study period ranged from 1910 to 2020 and included the following epidemics: Spanish flu (USA, 1918–1920) (one study), severe acute respiratory syndrome (Hong Kong and Taiwan, 2003) (five studies), human monkeypox (Nigeria, 2017) (one study), Ebola virus disease (Guinea, Uganda and Sierra Leone, 2000–2016) (four studies) and COVID-19 (Australia, Bangladesh, China, Denmark, France, Greece, India, Iran, Italy, Japan, South Korea, Taiwan, UK, USA and worldwide, 2019–2020) (45 studies), all of which were due to viral infections.

### Systematic review

#### Death by suicide

In the seven studies describing death by suicide (Table 1), there were two cohort studies, one case-control study and four ecological studies, which reported at least 167 suicides.

**Table 1.** Studies reporting death by suicides

Citation	Study design	Epidemic	Study group(s)	Main findings
Yip *et al*. (2010)	Case-control + qualitative	SARS; Hong Kong	(1) SARS-related suicides in older adults (*n* = 22). (2) 44 non-SARS-related suicides matched for age, gender and month	No difference between groups in terms of socioeconomic or illness burden. SARS-related suicides more likely to feature feeling disconnected (7/22, 32%) than non-SARS-related suicides (2/44, 5%), *p* = 0.02
Ogoina *et al*. (2019)	Cohort	HMPX; Nigeria	Confirmed and probable HMPX cases (*n* = 21)	1 suicide
Hewlett and Amola (2003)	Cohort + qualitative	EVD; Uganda	EVD survivors (*n* = 60)	1 suicide
Cheung *et al*. (2008)	Ecological	SARS; Hong Kong	(1) Older adult population (*n* not reported; estimate: 795 539). (2) Older adult population from 1993 to 2004	Elderly suicide rates in the month and year of the pandemic were significantly higher than in ten previous years. Elderly suicide rates were also higher in 2003 than in 2004 with an IRR of 1.18 (95% CI: 1.01–1.39, *p* = 0.039)
Chan *et al*. (2006)	Ecological	SARS; Hong Kong	Older adults (*n* not reported; estimate: 795 539).	Suicide rates were higher among the elderly in 2003 compared to 2002 (and a stable baseline for 4 previous years) with an IRR of 1.32 (95% CI: 1.11–1.57, *p* = 0.0019)
Wasserman (1992)	Ecological	Spanish Influenza; USA	(1) General population (*n* = 87 million). (2) Up to 75 million at other points in time	Suicide increased during the first phase of the pandemic but declined in the second phase
Isumi *et al*. (2020)	Ecological	COVID-19; Japan	(1) Children and adolescents under 20 during pandemic (*n* not reported). (2) Children and adolescents under 20 during 2 previous years (*n* not reported)	No significant change in suicide rates during school closure compared to previous years (IRR = 1.15, 95% CI: 0.81–1.64)

CI, confidence interval; COVID-19, coronavirus disease 2019; EVD, Ebola virus disease; HMPX, human monkeypox; IRR, incidence rate ratio; SARS, severe acute respiratory syndrome; USA, United States of America.

Four studies compared suicide incidence in epidemic and non-epidemic periods (Wasserman, 1992; Chan *et al*., 2006; Cheung *et al*., 2008; Isumi *et al*., 2020). One ecological study examining the impact of Spanish flu, World War I and the prohibition of alcohol on suicide in the general population found that all-cause mortality risks were positively correlated with death by suicide (Wasserman, 1992). The author noted that suicide increased after the first wave of the pandemic in 1919, but that a similar effect was not evident after the second wave (Wasserman, 1992). Two ecological studies examined the incidence of death by suicide among the elderly population in Hong Kong during the SARS epidemic. One study compared the year of the outbreak with the previous year, having shown stable suicide rates for four years prior to the outbreak, and found that suicides increased with an incidence rate ratio of 1.32 (95% CI: 1.11–1.57%) (Chan *et al*., 2006). The peak was in April, at the worst point of the epidemic. Further analysis found the increased rate was restricted to older women and did not affect younger age groups. A second study confirmed this peak using a more complete data set (due to delays in suicide reporting) and also found a trough in suicides two months later, eliminating a usual seasonal peak and suggesting that some suicides may have been brought forward by the epidemic (Cheung *et al*., 2008). The study also found that the rate of suicide in the year following the epidemic remained above the pre-pandemic levels, despite having declined from the previous year. One cohort study in Japan of children and adolescents under the age of 20 found no significant change in suicide rates during the period of pandemic-related school closure compared to previous years (Isumi *et al*., 2020).

One study compared suicides believed to be related to SARS to suicides unrelated to SARS from the same year (Yip *et al*., 2010). There were no differences between the groups in sociodemographic variables, history of psychiatric disorder, medical comorbidity or level of dependence on others, but feeling disconnected was more common in the individuals with SARS-related suicide. Among the suicides thought to be related to SARS, common problems were fear of infection, social isolation, disrupted routines and fear of being a burden to society.

Two small cohort studies each reported a single suicide in confirmed cases of Ebola virus disease (*n* = 60) and human monkeypox (*n* = 21) (Hewlett and Amola, 2003; Ogoina *et al*., 2019).

#### Self-harm

Of the nine studies describing self-harm summarised in Table 2, there were two cohort studies, two cross-sectional studies, and five ecological studies.

**Table 2.** Studies reporting self-harm

Citation	Study design	Epidemic	Study group(s)	Main findings
Iob *et al*. (2020)	Cross-sectional	COVID-19; UK	General population (*n* = 44 775)	2174 (4.9%) self-harmed during first month of national lockdown
Keita *et al*. (2017)	Cohort	EVD; Guinea	Infected individuals (*n* = 256)	3 (1.2%) attempted suicide
Hou *et al*. (2020)	Cross-sectional	COVID-19; China	Senior high school students (*n* = 859)	64 (7.5%) attempted suicide. Risk factors were being female and a poor academic record
Jefsen *et al*. (2020)	Cross-sectional	COVID-19; Denmark	Psychiatric patients with pandemic-related self-harm or suicidality (*n* = 74)	10 (13.5%) attempted suicide. 10 (13.5%) self-harmed
Olding *et al*. (2020)	Ecological	COVID-19; UK	(1) ED attendances with traumatic penetrating injuries (*n* = 30). (2) ED attendances with traumatic penetrating injuries from 2 previous years (*n* = 94)	Self-harm seemed to increase in absolute terms (1 case in 2018; 5 in 2019; 8 in 2020) and as a proportion of all penetrating trauma, but no testing for statistical significance conducted
Jacob *et al*. (2020)	Ecological	COVID-19; Australia	(1) Trauma admissions (*n* = 97). (2) Trauma admissions in previous 4 years (*n* = 557)	No significant difference in number of admissions for self-harm but numbers small
Pignon *et al*. (2020)	Ecological	COVID-19; France	(1) Emergency department psychiatric presentations (*n* = 553). (2) ED psychiatric presentations in previous year (*n* = 1224)	32 suicide attempts compared to 75 in previous year. No statistically significant difference
Hernández-Calle *et al*. (2020)	Ecological	COVID-19; USA	(1) ED attendances related to suicide (*n* not reported). (2) ED attendances related to suicide in 1 year prior to pandemic (*n* not reported)	Fewer suicide-related attendances for every week of the pandemic after first confirmed case of COVID-19 (*β* = −1.73, 95% CI: −0.90 to −2.56)
Huang *et al*. (2005)	Ecological	SARS; Taiwan	(1) ED attendees during pandemic (*n* = 17 586). (2) ED attendees during year prior to pandemic (*n* not stated)	3.3 (s.d. 1.8) patients presented each day having attempted suicide via drug overdose during peak epidemic, compared to 2.5 (s.d. 1.8) pre-epidemic and 2.3 (s.d. 1.4) post-epidemic (not statistically significant)

CI, confidence interval; COVID-19, coronavirus disease 2019; ED, emergency department; EVD, Ebola virus disease; SARS, severe acute respiratory syndrome; s.d., standard deviation; USA, United States of America.

Five studies provided comparative data for epidemic and non-epidemic populations, all of which examined emergency department attendances for self-harm (Huang *et al*., 2005; Hernández-Calle *et al*., 2020; Jacob *et al*., 2020; Olding *et al*., 2020; Pignon *et al*., 2020). One study during SARS examined attendances for suicide attempts via drug overdose, finding that on a background of reduced attendances overall and reduced attendances for psychiatric problems, in particular, attendances for overdose appeared to have increased, but this was not statistically significant (Huang *et al*., 2005). Three studies of the COVID-19 pandemic showed no evidence of a difference in numbers of attendances (Jacob *et al*., 2020; Olding *et al*., 2020; Pignon *et al*., 2020) and one showed a reduction (Hernández-Calle *et al*., 2020), though numbers tended to be small.

Of the studies that did not present comparative data for epidemic and non-epidemic populations, four studies reported that between 1.2% (95% CI: 0.4–3.4%) and 13.5% (95% CI: 7.5–23.1%) of individuals self-harmed over a time period of between 30 days and 24 months (Keita *et al*., 2017; Hou *et al*., 2020; Iob *et al*., 2020; Jefsen *et al*., 2020). In a cohort study of confirmed Ebola virus disease cases, three patients [1.2% (95% CI: 0.4–3.4%)] attempted suicide after discharge (Keita *et al*., 2017). During COVID-19, one large representative survey found that 4.9% (95% CI: 4.6–5.1%) of the general population in the United Kingdom self-harmed in the first month of lockdown (Iob *et al*., 2020). A much smaller sample of senior high school students in China during COVID-19 found that 7.5% (95% CI: 5.9–9.4%) had attempted suicide (Hou *et al*., 2020). The highest prevalence was in an enriched sample of 74 psychiatric patients in Denmark with COVID-19-related self-harm or suicidality, of whom 10 (13.5%) attempted suicide and 10 (13.5%) self-harmed (Jefsen *et al*., 2020).

One study examined risk factors for suicide attempt among high school children, finding that being female and having a poor academic record were associated with increased risk (Hou *et al*., 2020).

#### Thoughts of suicide or self-harm

In the 45 studies reporting data on thoughts of suicide or self-harm, as described in Table 3, there were six cohort studies, one case-control study, 30 cross-sectional studies, three ecological studies and five studies of internet search engine results.

**Table 3.** Studies reporting thoughts of suicide or self-harm

Citation	Study design	Epidemic	Study group(s)	Main findings
Iob *et al*. (2020)	Cohort	COVID-19; UK	General population (*n* = 44 775)	7984 (17.8%) had thoughts of suicide or self-harm during the first month of national lockdown
Kim *et al*. (2020)	Cohort	COVID-19; South Korea	Caregivers who were quarantining with young patients (*n* = 72)	3 (4.2%) experienced thoughts of suicide
Hao *et al*. (2020)	Cross-sectional	COVID-19; China	(1) Psychiatric patients with depression and anxiety (*n* = 76). (2) Healthy controls (*n* = 109)	Thoughts of suicide present in 12 (16%) of psychiatric patients and only 1 (0.9%) of healthy controls. Thoughts of suicide was more intense in psychiatric patients (*p* = 0.003)
Tan *et al*. (2020)	Cross-sectional	COVID-19; China	(1) Full-time employees returning to work in the epidemic (*n* = 673). Comparisons made between workers and technical staff (*n* = 551) and management and executive staff (*n* = 122)	Thoughts of suicide present in 11 (1.6%)
Kaparounaki *et al*. (2020)	Cross-sectional	COVID-19; Greece	University students (*n* = 1000)	97 (9.7%) currently considering suicide, which was higher than normative data for general population, but prior suicidal and self-harming behaviour frequency was also higher than normative data
Czeisler *et al*. (2020)	Cross-sectional	COVID-19; USA	General population (*n* = 5470)	585 (10.7%) had seriously considered suicide in the previous 30 days. Thoughts of suicide was more common among 18–24s, minority ethnic groups, unpaid care-givers and essential workers
Li *et al*. (2020)	Cross-sectional	COVID-19; Taiwan	General population (*n* = 1970)	212 (10.8%) experienced thoughts of suicide. Independent predictors for thoughts of suicide were young age, less handwashing, low perceived social support, lower COVID-19-related support and poorer self-reported physical health
Xin *et al*. (2020)	Cross-sectional	COVID-19; China	University students (*n* = 24 378)	3153 (12.9%) experiencing thoughts of suicide or self-harm. Mandatory quarantine significantly associated with thoughts of self-harm/suicide (OR = 4.98, *p* *<* *0*.001.)
Wang *et al*. (2020)	Cross-sectional	COVID-19; USA	University students (*n* = 1994)	366 (18.0%) had thoughts of suicide or self-harm
Cai *et al*. (2020)	Case-control	COVID-19; China	(1) Hospital staff managing COVID-19 (*n* = 1173). (2) Hospital staff not managing COVID-19, matched (*n* = 1173)	141 (12.0%) of frontline staff and 105 (9.0%) of non-frontline staff experienced thoughts of suicide. Crude OR = 1.39 (95% CI: 1.06–1.82); Adjusted OR = 1.25 (95% CI: 0.92–1.71)
Hamm *et al*. (2020)	Cohort	COVID-19; USA	(1) Older adults with treatment-resistant depression (*n* = 72). (2) Same individuals pre-pandemic	Thoughts of suicide or self-harm present in 7 (10%) during the pandemic and 7 (10%) pre-pandemic
Wu *et al*. (2020*a*)	Cohort	COVID-19; China	Infected individuals who had been discharged from hospital (*n* = 370)	4 (1.1%) experienced thoughts of suicide or self-harm
Bowen *et al*. (2016)	Cohort	EVD; Liberia	Infected individuals, recovered (*n =* 82)	2 (2.3%) experienced thoughts of suicide
Keita *et al*. (2017)	Cohort	EVD; Guinea	Infected individuals, recovered (*n* = 256, 33 of whom reviewed by psychiatrist)	Out of 11 cases with severe depression, 1 presented with thoughts of suicide
Hou *et al*. (2020)	Cross-sectional	COVID-19; China	Senior high school students (*n* = 859)	269 (31.3% had thoughts of suicide). Risk factors were being female, poor academic attainment and having no siblings
Ahorsu *et al*. (2020)	Cross-sectional	COVID-19; Iran	Pregnant women and their husbands (*n* = 580)	255 (44.0%) experienced thoughts of suicide or self-harm. Thoughts were associated with fear of COVID-19
Yang *et al*. (2020)	Cross-sectional	COVID-19; China	Young cancer patients (*n* = 197)	BSI score mean was 8.6 (s.d. 7.8). BSI was positively correlated with adverse childhood events, anxiety symptoms and CRP but negatively correlated with sleep quality
Fitzpatrick *et al*. (2020)	Cross-sectional	COVID-19; USA	General population (*n* = 10 368)	1820 (17.6%) were classified as high risk according to the SBQ-R. Score was higher in certain groups: ethnic minorities, families with children, unmarried, young
Gratz *et al*. (2020)	Cross-sectional	COVID-19; USA	Amazon MTurk Users/General population (*n* = 500)	Using a threshold of a score of 3 + on the DSI-SS, 58 (11.6%) were at high risk of suicide. Stay-at-home orders were associated with increased suicide risk, which seemed to be mediated by ‘thwarted belongingness’
Islam *et al*. (2020)	Cross-sectional	COVID-19; Bangladesh	Adult population (*n* = 340)	Thoughts of suicide associated with fear resulting from COVID-19 (*r* = 0.18, *p* < 0.01)
Patsali *et al*. (2020)	Cross-sectional	COVID-19; Greece	University students (*n* = 1535)	In those without a history of suicide attempt, suicidality increased in 38.7% of females and 37.6% of males. In those with a history of suicide attempt, suicidality increased in 52.5% of females and 43.8% of males
Xiaoming *et al*. (2020)	Cross-sectional	COVID-19; China	Hospital staff (*n* = 8817)	576 (6.5%) had thoughts of suicide/self-harm
Zhou *et al*. (2020)	Cross-sectional	COVID-19; China	(1) Frontline hospital staff (*n* = 606). (2) General population (*n* = 1099)	13.0% met threshold for suicide risk on the MINI. No significant difference between hospital staff and general population
Qian *et al*. (2020)	Cross-sectional	COVID-19; China	Infected individuals, acute illness (*n* = 106)	26 (24.5%) had thoughts of self-harm or suicide
Benatti *et al*. (2020)	Cross-sectional	COVID-19; Italy	Patients with OCD under tertiary psychiatric care (*n* = 123). Comparison between those whose OCD worsened and those did not	Thoughts of suicide occurred in 4 (3.3%), exclusively among those with a worsening in their OCD
Jefsen *et al*. (2020)	Cross-sectional	COVID-19; Denmark	Psychiatric patients with pandemic-related self-harm or suicidality (*n* = 74)	34 (45.9%) had thoughts of suicide. 14 (18.9%) had thoughts of self-harm. 13 (17.6%) had a passive wish to die of COVID-19
Killgore *et al*. (2020*c*)	Cross-sectional	COVID-19; USA	General population (*n* = 1013)	Thoughts of suicide or self-harm were significantly correlated with worry about COVID-19 and insomnia severity. Mediation analysis suggested that the intervening variable of insomnia severity accounted for the connection between COVID-19 worries and thoughts of suicide
Killgore *et al*. (2020*b*)	Cross-sectional	COVID-19; USA	General population (*n* = 1013)	Of 436 participants with high levels of loneliness, 152 (34.9%) had thoughts of suicide or self-harm, while among the 577 who were not lonely, only 26 (4.5%) had thoughts of suicide or self-harm (OR = 11.0, 95% CI: 7.0–17.1, *p* < 0.00001)
Killgore *et al*. (2020*a*)^a^	Cross-sectional	COVID-19; USA	Amazon MTurk Users/General population (*n* = 3120).	Thoughts of suicide/self-harm increased over the study period [178/1013 (17.6%) in April; 212/1037 (20.4%) in May; 248/1070 (23.2%) in June]. Thoughts became more frequent over the study period in groups that were under lockdown, but it remained at the same frequency among those not under lockdown
Killgore *et al*. (2020*e*)	Cross-sectional	COVID-19; USA	Amazon MTurk users/general population (*n* = 1004)	Lower scores for psychological resilience were associated with thoughts of suicide (*r* = −0.38, *p* < 0.00001)
Killgore *et al*. (2020*d*)	Cross-sectional	COVID-19; USA	Amazon MTurk users/general population (*n* = 3121)	Loneliness scores increased during the period and were positively correlated with thoughts of suicide (April *ρ* = 0.42, May *ρ* = 0.40 and June *ρ* = 0.39, *p* < 0.00001)
Lee (2020)	Cross-sectional	COVID-19; USA	Amazon MTurk uers/general population (*n* = 775)	Passive thoughts of suicide related to the pandemic was measured on a five-point Likert scale, where 0 = not at all and 4 = nearly every day. Mean score 1.6, s.d. 1.5.
Lee *et al*. (2020)	Cross-sectional	COVID-19; USA	Amazon MTurk Users/General population (*n* = 398)	Passive thoughts of suicide related to the pandemic was measured on a 5-point Likert scale, where 0 = not at all and 4 = nearly every day. Mean score 0.75, s.d. 1.18
Shen *et al*. (2020)	Cross-sectional	COVID-19; China	ICU nurses (*n* = 85)	Thoughts of suicide present in 2 (2.4%)
Sharif *et al*. (2020)	Cross-sectional	COVID-19; worldwide	Neurosurgeons (*n* = 375).	19 (5.1%) experienced thoughts of suicide
Sheng *et al*. (2005)	Cross-sectional	SARS; Hong Kong	Infected individuals (*n =* 102)	2 (2.0%) in the acute phase and 0 (0%) in the convalescent phase experienced thoughts of suicide
Secor *et al*. (2020)	Cross-sectional	EVD; Liberia, Sierra Leone & Guinea	Infected individuals, recovered (*n =* 145)	Thoughts of suicide/self-harm present in at least 10% in each country
Wu *et al*. (2020*b*)	Ecological	COVID-19; China	(1) Pregnant women during pandemic (*n* = 1285). (2) Pregnant women pre-pandemic (*n* = 2839)	Awareness of pandemic associated with increased risk of self-harm thoughts (aRR = 2.9, 95% CI: 1.7–8.9%, *p* = 0.005)
Titov *et al*. (2020)	Ecological	COVID-19; Australia	(1) Individuals undergoing psychological assessments during pandemic (*n* = 1334). (2) Individuals undergoing psychological assessments in the previous months (*n* = 1338)	Thoughts of suicide present in 367 (27.5%) during pandemic and in 423 (30.6%) previously. No evidence for difference (*χ*^2^ = 3.11, *p* = 0.08)
Smalley *et al*. (2020)	Ecological	COVID-19; USA	(1) Emergency department attendees during pandemic (*n* = 56 453). (2) Emergency department attendees in year prior to pandemic (*n* = 31 387)	Attendances due to thoughts of suicide fell from 1144 (2.03%) in 2019 to 451 (1.44%) in 2020
Rana (2020)	Search engine results	COVID-19; India	General population (*n* not reported)	Positive correlation between daily infection deaths reported and searches for ‘suicide’ (*r* = 0.20, *p* < 0.05)
Jacobson *et al*. (2020)	Search engine results	COVID-19; USA	General population (*n* not reported). Time series during pandemic	Suicide-related search queries increased rapidly prior to enactment of stay-at-home policies, followed by a levelling off after their introduction
Halford *et al*. (2020)	Search engine results	COVID-19; USA	(1) General population (*n* not reported). (2) General population prior to pandemic	Of the six specific suicide-related search terms,four were less common during the pandemic and for two there was no evidence of difference. Search terms related to some risk factors for suicide were elevated
Sinyor *et al*. (2020)	Search engine results	COVID-19; worldwide & USA	(1) General population (*n* not reported). (2) General population prior to pandemic (5 April 2015–2029 February 2020)	There were significant reductions in searches for the word ‘suicide’ both worldwide [−12% (95% CI: −22% to −1%)] and in the United States [−17% (95% CI: −28% to −4%)]. The same was observed for ‘suicide methods’ both worldwide [−39% (95% CI: −59% to −9%)] and in the United States [−36% (95% CI: −57% to −6%)]. Changes in searches for ‘how to commit suicide’ and ‘how to kill yourself’ were not statistically significant
Knipe *et al*. (2020)	Search engine results	COVID-19; worldwide, Italy, Spain, UK and USA	General population (*n* not reported)	Searching for topics related to suicide fell after the pandemic was declared. In the UK, USA and Italy, suicide-related searches started to fall as the number of COVID-19 deaths started to rise, but these increased again after lockdown was announced in each country

a Supplemented with additional information from the author.

aRR, adjusted risk ratio; BSI, Beck Suicide Ideation Scale; CI, confidence interval; COVID-19, coronavirus disease 2019; CRP, C-reactive protein; DSI-SS, Depression Symptom Index-Suicide Subscale; EVD, Ebola virus disease; ICU, intensive care unit; MINI, Mini International Neuropsychiatric Interview; MTurk, Mechanical Turk; OCD, Obsessive Compulsive Disorder; OR, odds ratio; SBQ-R, Suicide Behaviours Questionnaire-Revised; s.d., standard deviation; UK, United Kingdom; USA, United States of America.

Five studies reported comparative results for epidemic and non-epidemic populations, all of which studied the COVID-19 pandemic. The most generalisable was a large web-based survey of US populations which reported that 10.7% of respondents reported having seriously considered suicide in the previous 30 days, which was compared to similar survey data from two years prior indicating a comparable figure of 4.3% (Substance Abuse and Mental Health Services Administration, 2019; Czeisler *et al*., 2020). One study found that emergency department attendances with thoughts of suicide fell compared to the same period in the previous year (Smalley *et al*., 2020). Another study of individuals undergoing psychological assessments found no difference in the frequency of thoughts of suicide compared to individuals referred in the months prior to the epidemic (Titov *et al*., 2020). One small cohort study that followed elderly people with depression before and during the epidemic found no evidence of a difference in frequency of thoughts of suicide (Hamm *et al*., 2020). A study of pregnant women found that thoughts of self-harm were more common during the pandemic than prior to it (Wu *et al*., 2020*b*).

To consider the prevalence of thoughts of suicide and self-harm, we divided our studies by population into the general population, children or adolescents, health care workers, psychiatric patients, infected patients and recovered patients.

Studies of general populations found that reported frequency of thoughts of suicide or self-harm were present in between 0.9% (95% CI 0.0 to 5.0%) and 20.3% (95% CI 18.9 to 21.8%) over a time period of between 1 week and 2 weeks (Czeisler *et al*., 2020; Hao *et al*., 2020; Iob *et al*., 2020; Kaparounaki *et al*., 2020; Killgore *et al*., 2020*a*; Kim *et al*., 2020; Li *et al*., 2020; Tan *et al*., 2020; Wang *et al*., 2020; Xin *et al*., 2020). In children and adolescents, one study found thoughts of suicide to be present in 31.3% (95% CI: 28.3–34.5%) over a time period of 6 months (Hou *et al*., 2020). Among health care workers, thoughts of suicide or self-harm were found to be present in between 2.4% (95% CI: 0.3–8.2%) and 13.9%. (95% CI: 12.3–15.6%) over a time period of between 14 days and 30 days (Cai *et al*., 2020; Sharif *et al*., 2020; Shen *et al*., 2020; Xiaoming *et al*., 2020). Among patients with pre-existing mental illnesses, thoughts of suicide or self-harm occurred in between 9.1% (95% CI: 2.5–21.7%) and 27.5% (95% CI: 25.1–30.0%) over a time period of 14 days (Benatti *et al*., 2020; Hamm *et al*., 2020; Hao *et al*., 2020; Titov *et al*., 2020). Among infected individuals who were acutely unwell, thoughts of suicide or self-harm were present in between 2.0% (95% CI: 0.5–6.9%) (measured in retrospect) and 24.5% (95% CI: 16.7–33.8%) (measured contemporaneously) over a time period of 14 days (Sheng *et al*., 2005; Qian *et al*., 2020). Five studies examined individuals who had recovered from the epidemic infection, finding the frequency of thoughts of suicide or self-harm of between 0.0% (95% CI: 0.0–3.6%) and 15.7% (95% CI: 13.9–17.7%) over a time period of between ‘several days’ and a mean of 42 days (Sheng *et al*., 2005; Bowen *et al*., 2016; Keita *et al*., 2017; Secor *et al*., 2020; Wu *et al*., 2020*a*).

Risk factors identified for thoughts of suicide or self-harm in the general population were young age (Czeisler *et al*., 2020; Fitzpatrick *et al*., 2020; Li *et al*., 2020), ethnic minority background (Czeisler *et al*., 2020; Fitzpatrick *et al*., 2020), essential worker status (Czeisler *et al*., 2020), families with children (Fitzpatrick *et al*., 2020), being unmarried (Fitzpatrick *et al*., 2020), prior psychiatric disorder (Hao *et al*., 2020), poorer physical health (Li *et al*., 2020), current lockdown (Gratz *et al*., 2020; Killgore *et al*., 2020*a*), less social support (Li *et al*., 2020), lower psychological resilience (Killgore *et al*., 2020*e*), concern about COVID-19 (Ahorsu *et al*., 2020; Islam *et al*., 2020; Killgore *et al*., 2020*c*; Wu *et al*., 2020*b*), lower adherence to infection control guidance (Li *et al*., 2020), loneliness and (Killgore *et al*., 2020*b*, 2020*d*) insomnia (Killgore *et al*., 2020*c*). There was no evidence for the difference in the prevalence of thoughts of suicide when comparing hospital staff to the general population (Zhou *et al*., 2020), or when comparing frontline *v*. non-frontline health care staff (Cai *et al*., 2020). Among children, risk factors were being female, poor academic attainment and having no siblings (Hou *et al*., 2020).

Three studies considered the relationship between economic factors and outcomes. One found a weak positive correlation between a recent job loss and suicide risk (*r* = 0.12, *p* < 0.01) (Gratz *et al*., 2020). The other two presented period prevalences by income brackets. A large UK study found a higher prevalence in lower-income groups (28.2% in the lowest stratum compared to 12.1% in the highest) (Iob *et al*., 2020), whereas there was little difference in a large US survey with a tendency towards the opposite trend (9.9% in the lowest stratum compared to 11.6% in the highest) (Czeisler *et al*., 2020).

##### 5.3.1.1 Search engine results

Five studies assessed trends of searches for terms related to suicide as a proxy measure for thoughts of suicide using the search engine Google (Halford *et al*., 2020; Jacobson *et al*., 2020; Knipe *et al*., 2020; Rana, 2020; Sinyor *et al*., 2020). One study in the United Kingdom, United States and Italy examining data from January to March 2020 found that suicide-related searches fell as the number of COVID-19 deaths started to rise but increased again after the lockdown was announced in each country (Knipe *et al*., 2020). However, another study looking at data in the United States made comparisons between states and examined the relationship between frequency of suicide-related searches and enactment of official stay-at-home orders, finding that an increase in suicide-related searches prior to the enactment of orders levelled off once these were implemented (Jacobson *et al*., 2020). One study of data from India found that there was a positive but weak correlation between the daily number of COVID-19 death reports and suicide-related searches over 52 days (Rana, 2020). Two studies compared search frequency with a period prior to the pandemic, both of which found overall reductions in suicide-related search terms, although Halford *et al*., found that use of terms related to some known suicide risk factors, such as unemployment, was increased (Halford *et al*., 2020; Sinyor *et al*., 2020).

#### Quality assessment

Overall, across the 57 studies, the mean score on the Newcastle-Ottawa Scale was 3.0 (s.d. 2.0). In total, 42 studies (74%) were deemed of low quality, 9 (16%) of moderate quality and only 6 (11%) of high quality. In terms of the domains of the Newcastle-Ottawa Scale, the mean score was 1.9 out of a maximum score of 4 (47%) on the selection domain, 0.8 out of 2 (42%) on the comparability domain, and 0.7 out of 3 (24%) on the outcome domain. The Main weaknesses were a lack of demonstration of an outcome at baseline, inadequate follow-up duration and high rates of loss at follow-up. The quality assessment rating for each paper is provided in the online Supplementary Material (pp. 6–9).

### Meta-analysis

**Fig. 2.**
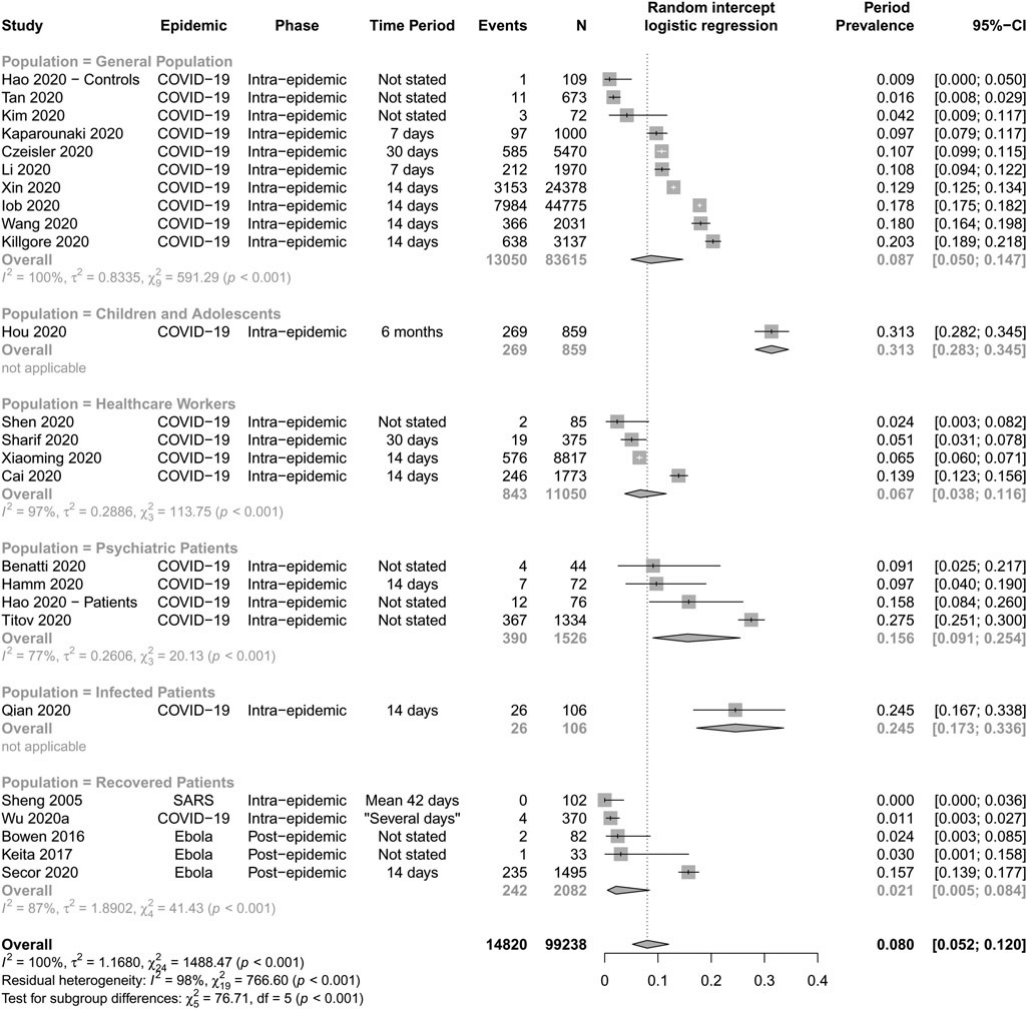
Forest plot for the period prevalence of thoughts of suicide or self-harm.

Meta-analysis was not possible for suicide or self-harm because results were not reported consistently between studies and actual numbers of events were frequently not available. For thoughts of suicide and self-harm, 24 studies contributed data on period prevalences with a total of 25 separate samples (see Fig. 2). These were separated into six distinct population subgroups (general population, children and adolescents, health care workers, psychiatric patients, infected patients and recovered patients). Overall, the period prevalence of thoughts of suicide and self-harm was 8.0% (95% CI: 5.2–12.0%; 14 820 of 99 238 cases in 24 studies) over a time period of between 7 days and 6 months. Among subgroups, prevalence was 8.7% (95% CI: 5.0–14.7%; 13 050 of 83 615 cases in ten studies) in the general population, 31.3% (95% CI: 28.3–34.5%; 269 of 859 cases in one study) in children and adolescents, 6.7% (95% CI: 3.8–11.6%; 843 of 11 050 cases in four studies) in health care workers, 15.6% (95% CI: 9.1–25.4%; 390 of 1526 cases in four studies) in psychiatric patients, 24.5% (95% CI: 17.3–33.6%; 26 of 106 cases in one study) in infected patients, and 2.1% (95% CI: 0.5–8.4%; 242 of 2082 cases in five studies) in recovered patients (see Fig. 2).

There were significant differences between certain population subgroups. Prevalence was significantly higher in children and adolescents compared to recovered patients (*p* < 0.001), psychiatric patients (*p* = 0.005), health care workers (*p* < 0.001) and the general population (*p* < 0.001). Prevalence was significantly higher in infected patients than in health care workers (*p* < 0.001), the general population (*p* = 0.003), and recovered patients (*p* < 0.001). Prevalence was significantly lower in recovered patients than in psychiatric patients (*p* = 0.023). There were no significant differences in prevalence between other subgroups (*p* > 0.05). There was one very high estimate, which was distinct in being the only study examining children and adolescents and in studying the longest time period (Hou *et al*., 2020). In a subgroup analysis by phase in the epidemic (Online Supplementary Fig. 7), only three studies were post-epidemic, while the rest were during an epidemic; there was no significant difference between these subgroups (*p* = 0.58) (online Supplementary material p. 23).

**Fig. 3.**
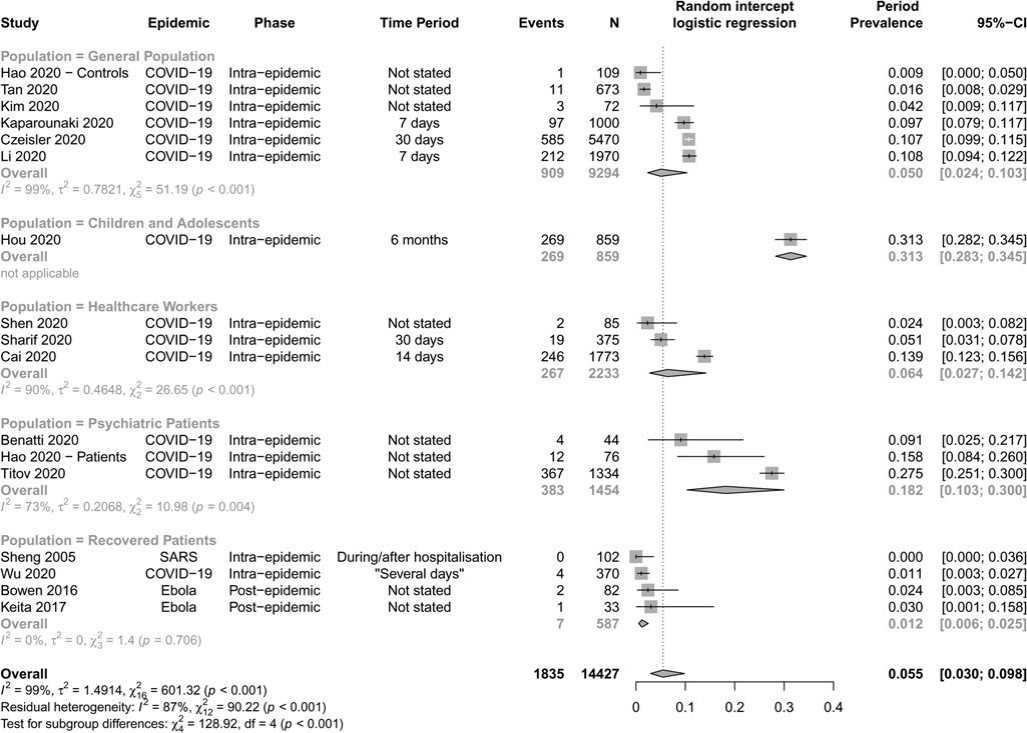
Forest plot for the period prevalence of thoughts of suicide.

Between-study heterogeneity was high (*I*^2^ = 100%, *p* < 0.001) and remained high when stratified by population subgroup (*I*^2^ = 98%, *p* < 0.001). Meta-regression of type of assessment showed a significantly higher period prevalence of thoughts of suicide or thoughts of self-harm (14.9%, 95% CI: 11.3–19.4%; 12 985 of 84 811 cases in eight studies) compared to thoughts of suicide alone (5.5%, 95% CI: 3.0–9.8%; 1835 of 14 427 cases in 16 studies). Studies describing only thoughts of suicide are shown in Fig. 3. Sensitivity analysis did not suggest that the meta-analytic estimate changed when removing any one study (online Supplementary Material pp. 17–21), but a sensitivity analysis did show a higher prevalence in the two moderate quality studies, compared to the others (all of which were of low quality) [10.7% (95% CI: 9.9–11.5%) *v*. 7.5% (95% CI 4.7–11.7%); *p* = 0.04] (online Supplementary material p. 22).

## Discussion

This study aimed to conduct a systematic review and meta-analysis of the literature on suicide, self-harm and thoughts of suicide or self-harm during infectious epidemics. We found little high-quality evidence comparing these outcomes to non-epidemic periods and the scope for generalisation is very limited. This work highlights the need for real-time monitoring of suicide, self-harm and thoughts of suicide or self-harm both during epidemics and in non-epidemic periods that can act as comparison groups.

In terms of death by suicide, studies of only two populations provide clear comparative evidence for the relationship between suicide and infectious epidemics, although both use an ecological design. The first describes an increase in suicides among the elderly in Hong Kong during SARS (Chan *et al*., 2006; Cheung *et al*., 2008), but this was restricted to women and did not extend to other age groups. The second examined suicide in Japan in those under the age of 20 and found no difference in frequency compared to previous years (Isumi *et al*., 2020).

In terms of self-harm, attendances to emergency departments showed no evidence of change compared to previous years in four studies (Huang *et al*., 2005; Jacob *et al*., 2020; Olding *et al*., 2020; Pignon *et al*., 2020), and a decrease in one study (Hernández-Calle *et al*., 2020), although numbers were generally small. Again, several studies found that a significant minority of individuals during an epidemic self-harmed, but the lack of comparison groups limits conclusions.

There is a greater quantity of evidence regarding thoughts of suicide and self-harm, although little of it provides a comparison to non-epidemic populations. One large US survey found that suicidal ideation was substantially more common than in previous years (Czeisler *et al*., 2020), as did a study of pregnant women (Wu *et al*., 2020*b*), but three studies of specific populations found no difference (Hamm *et al*., 2020; Smalley *et al*., 2020; Titov *et al*., 2020). Meta-analysis showed that overall the prevalence of thoughts of suicide or self-harm was 8.0% (95% CI: 5.2–12.0%) and prevalence of thoughts of suicide was 5.5% (95% CI: 3.0–9.8%) in those affected by an infectious epidemic, which is somewhat higher than the 12-month prevalence estimate of 2.0% (95% CI: 1.9–2.2%) from the WHO World Mental Health Surveys conducted in 21 countries (Borges *et al*., 2010). However, when the results were broken up into subgroups, differences emerged. Notably, one study of high-school students found higher rates of thoughts of suicide or self-harm than in the general population (Hou *et al*., 2020), although this is commonly the case outside of epidemics (Borges *et al*., 2010; McKinnon *et al*., 2016). There was also evidence from a single study that thought of suicide or self-harm may be common in infected patients (Qian *et al*., 2020). These results must be interpreted with caution, however, due to the diversity in measures used and the lack of head-to-head comparisons. In other subgroups that might be hypothesised to be at high risk (health care workers, recovered patients and psychiatric patients) we found no greater prevalence than in the general population. Moreover, it is established that only a minority of those with thoughts of suicide will attempt or die by suicide (Turecki and Brent, 2016).

Monitoring internet search engine terms related to suicide is an even more indirect measure of suicides and risks conflating increased interest in suicide secondary to media concerns with thoughts of suicide *per se*. It does, however, offer the promise of real-time monitoring of a population and studies have noted a longitudinal or geographical association between suicide-related search terms and death by suicide (Yang *et al*., 2011; Hagihara *et al*., 2012; Gunn and Lester, 2013; Barros *et al*., 2019). Although this has not been a universal finding (Sueki, 2011) to date, the data concerning COVID-19 suggest that at the level of day-by-day variation, there is a positive association between suicide-related search terms and the COVID-19 death rate (Jacobson *et al*., 2020; Knipe *et al*., 2020; Rana, 2020), but not when a larger time frame is examined (Halford *et al*., 2020; Sinyor *et al*., 2020).

Our study has several limitations, both in terms of the underlying evidence of the original articles and in the data synthesis. In terms of the original articles, in spite of the wealth of publicly available data on suicides globally, it was striking how little high-quality evidence was present in the peer-reviewed literature. The quality of studies was generally poor, with only 6 (11%) constituting high-quality evidence. The most common deficits were in the study outcomes, where follow-up was frequently inadequate and there were few studies that examined the years following an epidemic. Most studies focused on thoughts of suicide and self-harm, rather than death by suicide and self-harm and many studies relied on small samples. In addition, much of the data has been collected and reported whilst partway through a pandemic, giving an incomplete picture and not allowing longer-term follow-up. Some studies, particularly those relying on online surveys, are susceptible to selection bias because of variability in internet access and a tendency for completion rates to be related to demographic, financial and health-related outcomes of interest (Couper *et al*., 2007). Measurement bias is also likely since epidemics might change reporting practices for suicide, potentially resulting in under-reporting. The low quality of the majority of studies and the lack of control groups mean that our conclusions must be cautious. Our sensitivity analysis by study quality demonstrated that poor-quality studies may underestimate the prevalence of thoughts of suicide or self-harm. There are also issues with the generalisability of the results, given the high proportion of studies originating from China and the United States as well as a focus on quite specific subgroups. Interpretation of ecological studies risks conflating the exposure of a population with the exposure of individuals.

In terms of the process of conducting this systematic review, there were also inherent limitations, not least the extremely rapid growth of the literature, which more than doubled between the first and second database search. It is, therefore, impossible to be completely up-to-date, though we can discuss the different forms of data available, their contributions and their limitations. Our original protocol had to be adapted because it became apparent that some of our planned subgroup analyses would not be feasible because of lack of reporting of certain population characteristics and two of the eventual six subgroups only contained a single study each. Because original data were generally not available, our meta-analysis relied on aggregate – rather than an individual participant – data, which resulted in a loss of potentially interesting trends within studies. Very high heterogeneity between studies, which remained even after stratification by population subgroup, weakens the strength of any conclusions. Reasons for this heterogeneity likely include the populations studied, the period in question and the specific outcome measure. In particular, our results showed that the outcome used markedly affected prevalence figures for thoughts of suicide or self-harm, as studies that reported thoughts of suicide alone showed much lower estimates than those which also included thoughts of self-harm. The different time periods investigated in the various studies means that the pooled figures should be regarded with caution.

Our first conclusion must be that there is a substantial lack of evidence on the important and urgent question of whether the frequency of suicide, self-harm and thoughts of suicide or self-harm change during infectious epidemics. This is consistent with the findings of previous, less exhaustive reviews (Leaune *et al*., 2020; Zortea *et al*., 2020). The evidence that exists is generally of low quality and is inadequate to answer the relevant questions. There have been only two epidemics in two populations where robust data have been published in the peer-review literature examining the impact on death by suicide, finding that suicide was more frequent among the elderly during SARS and that there was no evidence of a difference in suicide frequency among children and adolescents during COVID-19 in Japan. However, more evidence is now starting to accumulate. Recent data from outside the search window of this systematic review in Norway and Australia have found no change in suicide rates during the COVID-19 pandemic compared to previous years (Knudsen *et al*., 2021; Leske *et al*., 2021). A Swedish study has recently found no correlation between influenza deaths over almost nine decades – including Spanish Flu – and a modest drop in suicides during the COVID-19 pandemic compared to the previous year (Rück *et al*., 2020).

Most of the available evidence suggests that the frequency of actual self-harm presentations to emergency departments does not change during a pandemic, but this is likely a small and unrepresentative sample of total self-harm. It is unclear whether thoughts of suicides change in prevalence during infectious epidemics. The largest study reviewed suggested a substantial increase in the United States (Czeisler *et al*., 2020), which is echoed by more recent data from the Czech Republic (Winkler *et al*., 2020). However, the findings from smaller studies were variable. Results from studies of internet search trends actually suggest a reduction in thoughts of suicides compared to non-epidemic periods. There was some evidence that certain groups, such as the young and ethnic minorities, may be at higher risk of thoughts of suicide. It is unclear to what extent evidence collected during previous epidemics may be relevant to the COVID-19 pandemic, as the global reach of COVID-19 and the relatively low case-fatality rate distinguishes it markedly from SARS and Ebola virus disease (Chan-Yeung and Xu, 2003; Kucharski and Edmunds, 2014; Rajgor *et al*., 2020).

The most urgent application of this study is for the development of up-to-date suicide estimates or even near real-time surveillance systems, which can inform policy making in the same way that a daily COVID-19 death toll does. There would be caveats to such data, as corrections may emerge at a later date, given difficulties in determining the cause of death in some cases. However, it is possible to undertake and UK data have already been presented, although not yet in peer-reviewed journals. These have shown that in several parts of England, there was no evidence of change in monthly suicides after the initiation of a lockdown (Appleby *et al*., 2020) and that nationally child suicides may have become more frequent, but this did not reach statistical significance (Odd *et al*., 2020). Second, existing national and international suicide data should be analysed to ascertain the relationship with past epidemics. Third, in the aftermath of the current pandemic, studies of the impact of suicide will be required with robust geographical, temporal and policy-related comparisons, investigating the impact of interventions such as lockdown on suicide. These will need to have a prolonged follow-up period, as the effects of the economic crisis on suicide have been shown to be delayed by up to several years (Iglesias-García *et al*., 2017). Fourth, studying thoughts of suicide may benefit from the timely use of electronic health apps. Fifth, reproducible and representative studies should be regularly conducted during non-epidemic periods to provide a point of comparison for subsequent studies. Lastly, in the context of suicide research, we note limitations on the use of certain measures – such as the PHQ-9 – that do not distinguish thoughts of suicide from thoughts of self-harm, as the information they provide may be too non-specific to be useful.

Beyond the need for further policy-driven research, there must be consideration of the potential changes in the numbers of suicides (mediated by unemployment, loneliness and reduced access to mental health services) in the models of the effects of efforts to control the pandemic. The media and policy-makers must avoid contributing to public alarm about suicide without sufficient evidence, given that data are so scarce on the subject; guidance for responsible reporting of suicides should be followed, including ensuring that suicides are not presented simplistically as caused solely by the current pandemic (Hawton *et al*., 2020; Independent Press Standards Organisation, 2020; Reger *et al*., 2020). As has previously been suggested, there are steps that policy-makers can take to reduce suicide that could have positive results far beyond the present pandemic (Moutier, 2020).

## Data Availability

Data extraction tables and *R* code will be made available to any interested parties on request to the corresponding author.

## Supplementary material

For supplementary material accompanying this paper visit http://dx.doi.org/10.1017/S2045796021000214. click here to view supplementary material
