# Suicide, self-harm and thoughts of suicide or self-harm in infectious disease epidemics: a systematic review and meta-analysis – CORRIGENDUM

**DOI:** 10.1017/S2045796021000354

**Published:** 2021-05-28

**Authors:** J. P. Rogers, E. Chesney, D. Oliver, N. Begum, A. Saini, S. Wang, P. McGuire, P. Fusar-Poli, G. Lewis, A. S. David

**Affiliations:** 1 Division of Psychiatry, University College London, London, UK; 2 South London and Maudsley NHS Foundation Trust, London, UK; 3 Department of Psychosis Studies, King's College London, London, UK; 4 GKT School of Medical Education, King's College London, London, UK; 5 Medical School, University College London, London, UK; 6 Department of Psychology, King's College London, London, UK; 7 Department of Brain and Behavioral Sciences, University of Pavia, Pavia, Italy; 8 UCL Institute of Mental Health, University College London, London, UK

In the above article an error was published in the Abstract. The sentence ‘The quality assessment found 42 studies were of high quality, nine of moderate quality and six of high quality.’ should read ‘The quality assessment found 42 studies were of low quality, nine of moderate quality and six of high quality.’

A revised abstract is below.

The author apologises for this error.

## Abstract

### Aims

Suicide accounts for 2.2% of all years of life lost worldwide. We aimed to establish whether infectious epidemics are associated with any changes in the incidence of suicide or the period prevalence of self-harm, or thoughts of suicide or self-harm, with a secondary objective of establishing the frequency of these outcomes.

### Methods

In this systematic review and meta-analysis, MEDLINE, Embase, PsycINFO and AMED were searched from inception to 9 September 2020. Studies of infectious epidemics reporting outcomes of (a) death by suicide, (b) self-harm or (c) thoughts of suicide or self-harm were identified. A random-effects model meta-analysis for the period prevalence of thoughts of suicide or self-harm was conducted.

### Results

In total, 1354 studies were screened with 57 meeting eligibility criteria, of which 7 described death by suicide, 9 by self-harm, and 45 thoughts of suicide or self-harm. The observation period ranged from 1910 to 2020 and included epidemics of Spanish Flu, severe acute respiratory syndrome, human monkeypox, Ebola virus disease and coronavirus disease 2019 (COVID-19). Regarding death by suicide, data with a clear longitudinal comparison group were available for only two epidemics: SARS in Hong Kong, finding an increase in suicides among the elderly, and COVID-19 in Japan, finding no change in suicides among children and adolescents. In terms of self-harm, five studies examined emergency department attendances in epidemic and non-epidemic periods, of which four found no difference and one showed a reduction during the epidemic. In studies of thoughts of suicide or self-harm, one large survey showed a substantial increase in period prevalence compared to non-epidemic periods, but smaller studies showed no difference. As a secondary objective, a meta-analysis of thoughts of suicide and self-harm found that the pooled prevalence was 8.0% overall (95% confidence interval (CI) 5.2–12.0%; 14 820 of 99 238 cases in 24 studies) over a time period of between seven days and six months. The quality assessment found 42 studies were of low quality, nine of moderate quality and six of high quality.

### Conclusions

There is little robust evidence on the association of infectious epidemics with suicide, self-harm and thoughts of suicide or self-harm. There was an increase in suicides among the elderly in Hong Kong during SARS and no change in suicides among young people in Japan during COVID-19, but it is unclear how far these findings may be generalised. The development of up-to-date self-harm and suicide statistics to monitor the effect of the current pandemic is an urgent priority.
