# Supplementary material for: Suicide, self-harm and thoughts of suicide or self-harm in infectious disease epidemics: a systematic review and meta-analysis
Source: Epidemiol Psychiatr Sci. 2021 Apr 27;30:e32. doi: 10.1017/S2045796021000214 (PMC7610720; doi:10.1017/S2045796021000214)

# **Appendix**

Completed suicide, self-harm and thoughts of suicide or self-harm in infectious disease epidemics: a systematic review and meta-analysis

Jonathan P Rogers MRCPsych, Edward Chesney MRCPsych, Dominic Oliver MSc, Nazifa Begum, Aman Saini, Simiao Wang, Prof Philip McGuire FMedSci, Paolo Fusar-Poli PhD, Prof Glyn Lewis PhD, Prof Anthony S David FMedSci

**eTable 1**: PRISMA checklist

**eTable 2**: Results of quality assessment

**eMethods 1**: Complete search strategy

**eFigures 1-5:** Leave-one-out sensitivity analyses for meta-analysis of thoughts of suicide or self-harm

**eFigure 6:** Sensitivity analysis by study quality for meta-analysis of thoughts of suicide or self-harm

**eFigure 7:** Subgroup analysis by phase in epidemic for meta-analysis of thoughts of suicide or self-harm

**eTable 1**: PRISMA checklist

| **eTable 1:** PRISMA guidelines for meta-analysis and systematic reviews | | | |
| --- | --- | --- | --- |
| **Section/topic** |   **#** | **Checklist item** |      **Page** |
| **TITLE** | | |  |
| Title |   1 | Identify the report as a systematic review, meta-analysis, or both. | 1 |
| **ABSTRACT** | | |  |
| Structured summary | 2 | Provide a structured summary including, as applicable: background; objectives; data sources; study eligibility criteria, participants, and interventions; study appraisal and synthesis methods; results; limitations; conclusions and implications of key findings; systematic review registration number. | 2-3 |
| **INTRODUCTION** | | |  |
| Rationale | 3 | Describe the rationale for the review in the context of what is already known. | 4 |
| Objectives | 4 | Provide an explicit statement of questions being addressed with reference to participants, interventions, comparisons, outcomes, and study design (PICOS). | 4 |
| **METHODS** | | |  |
| Protocol and registration |   5 | Indicate if a review protocol exists, if and where it can be accessed (e.g., Web address), and, if available, provide registration information including registration number. | 5 |
| Eligibility criteria | 6 | Specify study characteristics (e.g., PICOS, length of follow-up) and report characteristics (e.g., years considered, language, publication status) used as criteria for eligibility, giving rationale. | 5 |
| Information sources | 7 | Describe all information sources (e.g., databases with dates of coverage, contact with study authors to identify additional studies) in the search and date last searched. | 5 |
| Search | 8 | Present full electronic search strategy for at least one database, including any limits used, such that it could be repeated. | Table 1 & eMethods 1 |
| Study selection | 9 | State the process for selecting studies (i.e., screening, eligibility, included in systematic review, and, if applicable, included in the meta-analysis). | 5 |
| Data collection process | 10 | Describe method of data extraction from reports (e.g., piloted forms, independently, in duplicate) and any processes for obtaining and confirming data from investigators. | 5 |
| Data items | 11 | List and define all variables for which data were sought (e.g., PICOS, funding sources) and any assumptions and simplifications made. | 6 |
| Risk of bias in individual studies | 12 | Describe methods used for assessing risk of bias of individual studies (including specification of whether this was done at the study or outcome level), and how this information is to be used in any data synthesis. | 6 |
| Summary measures | 13 | State the principal summary measures (e.g., risk ratio, difference in means). | 6 |
| Synthesis of results | 14 | Describe the methods of handling data and combining results of studies, if done, including measures of consistency (e.g., *I*2) for each meta-analysis. | 6 |
| Risk of bias across studies | 15 | Specify any assessment of risk of bias that may affect the cumulative evidence (e.g., publication bias, selective reporting within studies). | 6 |
| Additional analyses | 16 | Describe methods of additional analyses (e.g., sensitivity or subgroup analyses, meta-regression), if done, indicating which were pre-specified. | 6 |
| **RESULTS** |  |  |  |
| Study selection | 17 | Give numbers of studies screened, assessed for eligibility, and included in the review, with reasons for exclusions at each stage, ideally with a flow diagram. | Figure 1 |
| Study characteristics | 18 | For each study, present characteristics for which data were extracted (e.g., study size, PICOS, follow-up period) and provide the citations. | Tables 2, 3, 4 |
| Risk of bias within studies | 19 | Present data on risk of bias of each study and, if available, any outcome level assessment (see item 12). | eTable 2 |
| Results of individual studies | 20 | For all outcomes considered (benefits or harms), present, for each study: (a) simple summary data for each intervention group (b) effect estimates and confidence intervals, ideally with a forest plot. | Figure 2 |
| Synthesis of results | 21 | Present results of each meta-analysis done, including confidence intervals and measures of consistency. | Figure 2 |
| Risk of bias across studies | 22 | Present results of any assessment of risk of bias across studies (see Item 15). | eTable 2 |
| Additional analysis | 23 | Give results of additional analyses, if done (e.g., sensitivity or subgroup analyses, meta-regression [see Item 16]). | eFigures 1-5 |
| **DISCUSSION** |  |  |  |
| Summary of evidence |   24 | Summarize the main findings including the strength of evidence for each main outcome; consider their relevance to key groups (e.g., healthcare providers, users, and policy makers). | 10-11 |
| Limitations | 25 | Discuss limitations at study and outcome level (e.g., risk of bias), and at review-level (e.g., incomplete retrieval of identified research, reporting bias). | 11 |
| Conclusions | 26 | Provide a general interpretation of the results in the context of other evidence, and implications for future research. | 11-12 |
| **FUNDING** |  |  |  |
| Funding | 27 | Describe sources of funding for the systematic review and other support (e.g., supply of data); role of funders for the systematic review. | 2 |

**eTable 2**: Results of quality assessment

| Number | First author | Publication Date | Title |  |
| --- | --- | --- | --- | --- |
| 1 | Killgore | 2020 | Suicidal ideation during the COVID-19 pandemic: The role of insomnia. | Low |
| 2 | Killgore | 2020 | Loneliness: A signature mental health concern in the era of COVID-19. | Low |
| 3 | Sharif | 2020 | COVID 19-Depression and Neurosurgeons. | Low |
| 4 | Hao | 2020 | Do psychiatric patients experience more psychiatric symptoms during COVID-19 pandemic and lockdown? A case-control study with service and research implications for immunopsychiatry. | Low |
| 5 | Tan | 2020 | Is returning to work during the COVID-19 pandemic stressful? A study on immediate mental health status and psychoneuroimmunity prevention measures of Chinese workforce. | Low |
| 6 | Sinyor | 2020 | Changes in Suicide and Resilience-related Google Searches during the Early Stages of the COVID-19 Pandemic. | Medium |
| 7 | Rana | 2020 | Elderly Suicides in India: An Emerging Concern during COVID-19 Pandemic. | Low |
| 8 | Wu | 2020 | Perinatal depressive and anxiety symptoms of pregnant women along with COVID-19 outbreak in China. | Medium |
| 9 | Jacobson | 2020 | Flattening the Mental Health Curve: COVID-19 Stay-at-Home Orders Are Associated With Alterations in Mental Health Search Behavior in the United States. | Low |
| 10 | Lee | 2020 | Coronavirus Anxiety Scale: A brief mental health screener for COVID-19 related anxiety. | Low |
| 11 | Huang | 2005 | Impact of severe acute respiratory syndrome (SARS) outbreaks on the use of emergency department medical resources. | High |
| 12 | Keita | 2017 | Depressive symptoms among survivors of Ebola virus disease in Conakry (Guinea): preliminary results of the PostEboGui cohort. | Low |
| 13 | Yip | 2010 | The impact of epidemic outbreak: the case of severe acute respiratory syndrome (SARS) and suicide among older adults in Hong Kong. | Medium |
| 14 | Cheung | 2008 | A revisit on older adults suicides and Severe Acute Respiratory Syndrome (SARS) epidemic in Hong Kong. | High |
| 15 | Chan | 2006 | Elderly suicide and the 2003 SARS epidemic in Hong Kong. | High |
| 16 | Wasserman | 1992 | The impact of epidemic, war, prohibition and media on suicide: United States, 1910-1920. | High |
| 17 | Shen | 2020 | Psychological stress of ICU nurses in the time of COVID-19. | Low |
| 18 | Ogoina | 2019 | The 2017 monkeypox outbreak in Nigeria-report of outbreak experience and response in the Niger Delta University Teaching Hospital, Bayelsa State, Nigeria. | Low |
| 19 | Hewlett | 2003 | Cultural Contexts of Ebola in Northern Uganda | Low |
| 20 | Bowen | 2016 | Survivors of Ebola virus disease have persistent neurological deficits (S53. 003) | Low |
| 21 | Sheng | 2005 | The effects of disease severity, use of corticosteroids and social factors on neuropsychiatric complaints in severe acute respiratory syndrome (SARS) patients at acute and convalescent phases | Low |
| 22 | Secor | 2020 | Mental health among Ebola survivors in Liberia, Sierra Leone and Guinea: results from a cross-sectional study | Low |
| 23 | Killgore | 2020 | Trends in suicidal ideation over the first three months of COVID-19 lockdowns | Low |
| 24 | Killgore | 2020 | Psychological resilience during the COVID-19 lockdown | Low |
| 25 | Killgore | 2020 | Three months of loneliness during the COVID-19 lockdown | Low |
| 26 | Halford | 2020 | Google searches for suicide and suicide risk factors in the early stages of the COVID-19 pandemic. | Low |
| 27 | Yang | 2020 | The effect and mechanism of adverse childhood experience on suicide ideation in young cancer patients during coronavirus disease 2019 (Covid-19) pandemic. | Low |
| 28 | Olding | 2020 | Penetrating trauma during a global pandemic: Changing patterns in interpersonal violence, self-harm and domestic violence in the Covid-19 outbreak. | Medium |
| 29 | Iob | 2020 | Abuse, self-harm and suicidal ideation in the UK during the COVID-19 pandemic. | Medium |
| 30 | Wang | 2020 | Investigating College Students' Mental Health During the COVID-19 Pandemic: An Online Survey Study. | Low |
| 31 | Zhou | 2020 | The prevalence and risk factors of psychological disturbances of frontline medical staff in china under the COVID-19 epidemic: Workload should be concerned. | Low |
| 32 | Isumi | 2020 | Do suicide rates in children and adolescents change during school closure in Japan? The acute effect of the first wave of COVID-19 pandemic on child and adolescent mental health. | High |
| 33 | Ahorsu | 2020 | Associations Between Fear of COVID-19, Mental Health, and Preventive Behaviours Across Pregnant Women and Husbands: An Actor-Partner Interdependence Modelling. | Low |
| 34 | Jacob | 2020 | Impact of societal restrictions and lockdown on Trauma admissions during the COVID-19 pandemic: A single centre cross-sectional observational study. | Medium |
| 35 | Patsali | 2020 | University students' changes in mental health status and determinants of behavior during the COVID-19 lockdown in Greece. | Low |
| 36 | Hou | 2020 | Prevalence of and factors associated with mental health problems and suicidality among senior high school students in rural China during the COVID-19 outbreak. | Low |
| 37 | Hernandez-Calle | 2020 | Trends in Psychiatric Emergency Department Visits Due to Suicidal Ideation and Suicide Attempts During the COVID-19 Pandemic in Madrid, Spain. | Medium |
| 38 | Islam | 2020 | Exploring COVID-19 stress and its factors in Bangladesh: A perception-based study. | Low |
| 39 | Xiaoming | 2020 | The psychological status of 8817 hospital workers during COVID-19 Epidemic: A cross-sectional study in Chongqing. | Low |
| 40 | Benatti | 2020 | What Happened to Patients With Obsessive Compulsive Disorder During the COVID-19 Pandemic? A Multicentre Report From Tertiary Clinics in Northern Italy. | Low |
| 41 | Jefsen | 2020 | COVID-19-related self-harm and suicidality among individuals with mental disorders. | Low |
| 42 | Cai | 2020 | The mental health of frontline and non-frontline medical workers during the coronavirus disease 2019 (COVID-19) outbreak in China: A case-control study. [References]. | Low |
| 43 | Xin | 2020 | Negative cognitive and psychological correlates of mandatory quarantine during the initial COVID-19 outbreak in China. [References]. | Low |
| 44 | Czeisler | 2020 | Mental Health, Substance Use, and Suicidal Ideation During the COVID-19 Pandemic - United States, June 24-30, 2020. | Medium |
| 45 | Li | 2020 | COVID-19-Related Factors Associated with Sleep Disturbance and Suicidal Thoughts among the Taiwanese Public: A Facebook Survey. | Low |
| 46 | Knipe | 2020 | Mapping population mental health concerns related to COVID-19 and the consequences of physical distancing: a Google trends analysis. | Low |
| 47 | Hamm | 2020 | Experiences of american older adults with pre-existing depression during the beginnings of the covid-19 pandemic: A multicity, mixed-methods study. | High |
| 48 | Gratz | 2020 | Thwarted belongingness and perceived burdensomeness explain the associations of covid-19 social and economic consequences to suicide risk. | Low |
| 49 | Fitzpatrick | 2020 | How bad is it? Suicidality in the middle of the covid-19 pandemic. | Low |
| 50 | Pignon | 2020 | Dramatic reduction of psychiatric emergency consultations during lockdown linked to COVID-19 in Paris and suburbs | Low |
| 51 | Smalley | 2020 | The impact of COVID-19 on suicidal ideation and alcohol presentations to emergency departments in a large healthcare system | Low |
| 52 | Titov | 2020 | Rapid report: Early demand, profiles and concerns of mental health users during the coronavirus (COVID-19) pandemic | Medium |
| 53 | Kaparounakia | 2020 | University students' mental health amidst the COVID-19 quarantine in Greece | Low |
| 54 | Lee | 2020 | Clinically significant fear and anxiety of COVID-19: A psychometric examination of the Coronavirus Anxiety Scale | Low |
| 55 | Wu | 2020 | Mental health status and related influencing factors of COVID-19 survivors in Wuhan, China | Low |
| 56 | Qian | 2020 | Investigation of the mental health of patients with novel coronavirus pneumonia | Low |
| 57 | Kim | 2020 | Psychological Impact of Quarantine on Caregivers at a Children's Hospital for Contact with Case of COVID-19. | Low |

**eMethods 1**: Complete search strategy

1. Search the following databases from inception:
   1. MEDLINE
   2. EMBASE
   3. PsycINFO
   4. AMED
2. Examine the reference lists of other review articles
3. Contact relevant experts in the field for further data

## **MEDLINE**

1. (epidemic or pandemic or outbreak or plague).mp. [mp=abstract, heading words, title]

2. exp Epidemics/

3. exp Pandemics/

4. exp Disease Outbreaks/

5. exp Plague/

6. 1 or 2 or 3 or 4 or 5

7. Infections/

8. exp Fever/

9. exp Viruses/

10. exp Bacteria/

11. exp Fungi/

12. exp Parasites/

13. exp Amoeba/

14. exp Amebiasis/

15. exp Bacterial Infections/

16. exp Parasitic Diseases/

17. exp Arenaviridae/ or exp Arenavirus/ or exp Arenaviridae Infections/

18. exp Botulism/

19. exp Poxviridae/ or exp Poxviridae Infections/

20. exp Chagas Disease/

21. exp Chikungunya virus/ or exp Chikungunya Fever/

22. exp Cholera/

23. exp Coronavirus/ or exp Coronavirus Infections/

24. exp SARS Virus/

25. exp Severe Acute Respiratory Syndrome/

26. exp Campylobacter/ or exp Campylobacter Infections/

27. exp Corynebacterium/ or exp Corynebacterium Infections/

28. exp Diphtheria/

29. exp Dracunculiasis/

30. exp Dracunculus Nematode/

31. exp Dysentery/

32. exp Ebolavirus/ or exp Hemorrhagic Fever, Ebola/ or exp Hemorrhagic Fevers, Viral/

33. exp Escherichia coli/

34. exp Flavobacteriaceae/

35. exp Encephalitis/

36. exp Enterovirus/

37. exp Flavivirus Infections/ or exp Flavivirus/

38. exp Francisella tularensis/ or exp Francisella/

39. exp Neisseria gonorrhoeae/

40. exp Gonorrhea/ or exp Neisseria gonorrhoeae/

41. exp Hantavirus/ or exp Hantavirus Infections/

42. exp "Hand, Foot and Mouth Disease"/

43. exp Hepatitis/

44. exp HIV/ or exp HIV Infections/

45. exp Acquired Immunodeficiency Syndrome/

46. exp Influenza, Human/

47. exp Lassa virus/ or exp Lassa Fever/

48. exp Leishmania/

49. exp Leishmaniasis/

50. exp Leprosy/

51. exp Mycobacterium leprae/

52. exp Leptospirosis/

53. exp Leptospira/

54. exp Legionella pneumophila/ or exp Legionnaires' Disease/

55. exp Listeria/

56. exp Listeriosis/

57. exp Malaria/

58. exp Plasmodium/

59. exp Marburg Virus Disease/ or exp Marburgvirus/

60. exp Meningitis/

61. exp Neisseria meningitidis/

62. exp Mumps/ or exp Mumps virus/

63. exp Mycobacterium/

64. exp Neisseria/

65. exp Measles/ or exp Measles Vaccine/

66. exp Monkeypox virus/ or exp Monkeypox/

67. exp Middle East Respiratory Syndrome Coronavirus/

68. exp Myocarditis/

69. exp Bunyaviridae Infections/

70. exp Plague/

71. exp Phlebovirus/

72. exp Pseudomonas/

73. exp Poliomyelitis/

74. exp Rabies/ or exp Rabies virus/

75. exp Rift Valley Fever/

76. exp Salmonella/ or exp Salmonella Infections/

77. exp Encephalopathy, Bovine Spongiform/ or exp Prion Diseases/ or exp Prions/

78. exp Creutzfeldt-Jakob Syndrome/

79. exp Scarlet Fever/

80. exp Seoul virus/

81. exp Shigella/

82. exp Smallpox/

83. exp Streptococcus/

84. exp Streptococcal Infections/

85. exp Staphylococcus/

86. exp Staphylococcal Infections/

87. exp Trypanosoma/

88. exp Trypanosomiasis/

89. exp Toxoplasma/

90. exp Toxoplasmosis/

91. exp Tuberculosis/

92. exp Tularemia/

93. exp Typhoid Fever/

94. exp Typhus, Epidemic Louse-Borne/

95. exp Rickettsia/

96. exp Vibrio/

97. exp West Nile virus/

98. exp Whooping Cough/

99. exp Yersinia/

100. exp Zika Virus Infection/ or exp Zika Virus/

101. (infectio* or fever or viral or virus or bacteria* or fung* or mycosis or mycoses or parasit* or amoeb* or ameb* or arenavir* or botulism or buffalopox or chagas or chikungunya or cholera* or coronavirus or COVID* or SARS* or "acute respiratory syndrome" or campylobacter or corynebacteri* or dengue or diphtheri* or dracunculiasis or "guinea worm" or dysenter* or ebola or "e. coli" or escherichia or elizabethkingia or encephaliti* or enterovir* or flavivirus or francisella or gonococc* or gonorrh* or hantavir* or "hand foot and mouth" or HFMD or orthohantavir* or hepatitis or HIV or AIDS or "human immunodeficiency virus" or "acquired immune deficiency syndrome" or influenza or flu or H1N1 or H1N2 or H2N2 or H3N2 or H3N8 or H5N1 or H7N2 or H7N3 or H7N7 or H9N2 or H10N7 or lassa or leishmania* or leprosy or leptospir* or Weil or legionn* or listeri* or malaria or plasmodium or Marburg or meningiti* or meningococc* or mumps or mycobacteri* or Neisseria or measles or monkeypox or morbilli or MERS or MERS-CoV or "Middle East respiratory syndrome" or myocarditi* or "o'nyong'nyong" or oropouche or pertussis or plague or bubonic or phlebovirus or pneumonic or pseudomonas or aeruginosa or polio* or "rabbit fever" or rabies or rickettsia or "rift valley fever" or salmonell* or "spongiform encephalopathy" or BSE or "mad cow disease" or Creutzfeld-Jakob or "vCJD" or "scarlet fever" or "Seoul orthohantavirus" or "Seoul virus" or SEOV or shigell* or smallpox or streptococc* or staphylococc* or trypanosom* or "sleeping sickness" or toxoplasm* or tuberculo* or TB or tularaemia or tularemia or typhoid or typhus or vibrio or "west nile" or "whooping cough" or yersinia or zika).mp. [mp=abstract, heading words, title]

102. 7 or 8 or 9 or 10 or 11 or 12 or 13 or 14 or 15 or 16 or 17 or 18 or 19 or 20 or 21 or 22 or 23 or 24 or 25 or 26 or 27 or 28 or 29 or 30 or 31 or 32 or 33 or 34 or 35 or 36 or 37 or 38 or 39 or 40 or 41 or 42 or 43 or 44 or 45 or 46 or 47 or 48 or 49 or 50 or 51 or 52 or 53 or 54 or 55 or 56 or 57 or 58 or 59 or 60 or 61 or 62 or 63 or 64 or 65 or 66 or 67 or 68 or 69 or 70 or 71 or 72 or 73 or 74 or 75 or 76 or 77 or 78 or 79 or 80 or 81 or 82 or 83 or 84 or 85 or 86 or 87 or 88 or 89 or 90 or 91 or 92 or 93 or 94 or 95 or 96 or 97 or 98 or 99 or 100 or 101

103. (suicid* or "self mutilat*" or "self-mutilat*" or selfmutilat* or "self harm" or "self-harm" or selfharm or "self injur*" or "self-injur*" or selfinjur* or "overdos*" or "self inflict*" or self-inflict* or selfinflict* or "auto mutilat*" or automutilat* or automutilat* or selfcutt* or self-cutt* or "self cutt*" or "head bang*" or headbang* or head-bang* or "self destruct*" or "selfdestruct*" or "self-destruct*" or "self immolat*" or self-immolat* or selfimmolat* or "self poison*" or self-poison* or selfpoison* or parasuicid*).mp. [mp=abstract, heading words, title]

104. exp suicide/ or exp suicidal ideation/ or exp suicide, attempted/ or exp suicide, completed/

105. exp Self Mutilation/

106. exp Self-Injurious Behavior/

107. exp Drug Overdose/

108. 103 or 104 or 105 or 106 or 107

109. 6 and 102 and 108

## **EMBASE**

1. exp epidemic/

2. exp pandemic/

3. plague/

4. (epidemic or pandemic or outbreak or plague).mp. [mp=abstract, heading words, title]

5. 1 or 2 or 3 or 4

6. exp infection/

7. exp fever/

8. exp virus/

9. exp virus infection/

10. exp bacterium/

11. exp bacterial infection/

12. exp fungus/

13. exp mycosis/

14. exp parasite/

15. exp parasitosis/

16. exp "amoeba (life cycle stage)"/

17. exp amebiasis/

18. exp Arenaviridae/

19. exp botulism/

20. exp Orthopoxvirus/

21. exp Chagas disease/

22. exp Chikungunya virus/ or exp chikungunya/

23. exp cholera toxin-induced diarrhea/ or exp cholera/

24. exp Coronavirinae/

25. exp Coronavirinae/

26. exp severe acute respiratory syndrome/

27. exp Campylobacter enteritis/ or exp Campylobacter/

28. exp Corynebacterium/

29. exp dengue/ or exp Dengue virus/

30. exp diphtheria/

31. exp dracunculiasis/

32. exp dysentery/

33. exp Ebola hemorrhagic fever/

34. exp Escherichia coli/

35. exp Elizabethkingia meningoseptica/ or exp Elizabethkingia/

36. exp encephalitis/

37. exp Enterovirus infection/ or exp Enterovirus/

38. exp Flavivirus infection/ or exp Flavivirus/

39. exp Francisella/

40. exp Neisseria/

41. exp gonorrhea/

42. exp Hantavirus infection/ or exp Hantavirus/

43. exp "hand foot and mouth disease"/

44. hepatitis/ or exp hepatitis virus/ or exp virus hepatitis/

45. exp Human immunodeficiency virus/

46. exp acquired immune deficiency syndrome/

47. influenza/

48. exp Lassa virus/ or exp Lassa fever/

49. exp Leishmania/

50. exp leprosy/

51. exp leptospirosis/

52. exp legionnaire disease/

53. exp Legionella/

54. exp Listeria/

55. exp listeriosis/

56. exp malaria/

57. exp Plasmodium/

58. exp Marburgvirus/ or exp virus hemorrhagic fever/ or exp Marburg hemorrhagic fever/ or exp Marburg virus/

59. exp meningitis/

60. exp meningococcemia/ or exp meningococcosis/ or exp Neisseria meningitidis/

61. exp Mumps virus/ or exp mumps/

62. exp Mycobacterium/

63. exp Measles virus/ or exp measles/

64. exp monkeypox/ or exp Monkeypox virus/

65. exp Middle East respiratory syndrome coronavirus/ or exp Middle East respiratory syndrome/

66. exp myocarditis/

67. exp O nyong nyong virus/ or exp O nyong nyong fever/

68. exp Orthobunyavirus/ or exp Oropouche virus/ or exp Bunyavirus/ or exp oropouche bunyavirus/

69. exp Bordetella pertussis/ or exp pertussis/

70. exp plague/

71. exp Yersinia pestis/

72. exp Phlebovirus infection/ or exp Phlebovirus/

73. exp Pseudomonas/

74. exp poliomyelitis/

75. exp tularemia/

76. rabies/ or exp Rabies virus/

77. exp Rickettsia/

78. exp Rift Valley fever virus/ or exp Rift Valley fever/ or exp Rift Valley fever bunyavirus/

79. exp Salmonella/

80. exp salmonellosis/

81. exp brain spongiosis/

82. exp bovine spongiform encephalopathy/

83. exp Creutzfeldt Jakob disease/

84. exp scarlet fever/

85. exp Seoul virus/

86. exp Shigella/

87. exp shigellosis/

88. exp smallpox/ or exp Smallpox virus/

89. exp Streptococcus/

90. exp Streptococcus infection/

91. exp Staphylococcus infection/ or exp Staphylococcus/

92. exp trypanosomiasis/

93. exp Trypanosoma/

94. exp Toxoplasma/

95. exp toxoplasmosis/

96. exp tuberculosis/

97. exp tularemia/

98. exp typhoid fever/

99. exp typhus/

100. exp Vibrio/

101. exp West Nile fever/ or exp West Nile flavivirus/ or exp West Nile virus/

102. exp Zika virus/ or exp Zika fever/

103. (infectio* or fever or viral or virus or bacteria* or fung* or mycosis or mycoses or parasit* or amoeb* or ameb* or arenavir* or botulism or buffalopox or chagas or chikungunya or cholera* or coronavirus or COVID* or SARS* or "acute respiratory syndrome" or campylobacter or corynebacteri* or dengue or diphtheri* or dracunculiasis or "guinea worm" or dysenter* or ebola or "e. coli" or escherichia or elizabethkingia or encephaliti* or enterovir* or flavivirus or francisella or gonococc* or gonorrh* or hantavir* or "hand foot and mouth" or HFMD or orthohantavir* or hepatitis or HIV or AIDS or "human immunodeficiency virus" or "acquired immune deficiency syndrome" or influenza or flu or H1N1 or H1N2 or H2N2 or H3N2 or H3N8 or H5N1 or H7N2 or H7N3 or H7N7 or H9N2 or H10N7 or lassa or leishmania* or leprosy or leptospir* or Weil or legionn* or listeri* or malaria or plasmodium or Marburg or meningiti* or meningococc* or mumps or mycobacteri* or Neisseria or measles or monkeypox or morbilli or MERS or MERS-CoV or "Middle East respiratory syndrome" or myocarditi* or "o'nyong'nyong" or oropouche or pertussis or plague or bubonic or phlebovirus or pneumonic or pseudomonas or aeruginosa or polio* or "rabbit fever" or rabies or rickettsia or "rift valley fever" or salmonell* or "spongiform encephalopathy" or BSE or "mad cow disease" or Creutzfeld-Jakob or "vCJD" or "scarlet fever" or "Seoul orthohantavirus" or "Seoul virus" or SEOV or shigell* or smallpox or streptococc* or staphylococc* or trypanosom* or "sleeping sickness" or toxoplasm* or tuberculo* or TB or tularaemia or tularemia or typhoid or typhus or vibrio or "west nile" or "whooping cough" or yersinia or zika).mp. [mp=abstract, heading words, title]

104. 6 or 7 or 8 or 9 or 10 or 11 or 12 or 13 or 14 or 15 or 16 or 17 or 18 or 19 or 20 or 21 or 22 or 23 or 24 or 25 or 26 or 27 or 28 or 29 or 30 or 31 or 32 or 33 or 34 or 35 or 36 or 37 or 38 or 39 or 40 or 41 or 42 or 43 or 44 or 45 or 46 or 47 or 48 or 49 or 50 or 51 or 52 or 53 or 54 or 55 or 56 or 57 or 58 or 59 or 60 or 61 or 62 or 63 or 64 or 65 or 66 or 67 or 68 or 69 or 70 or 71 or 72 or 73 or 74 or 75 or 76 or 77 or 78 or 79 or 80 or 81 or 82 or 83 or 84 or 85 or 86 or 87 or 88 or 89 or 90 or 91 or 92 or 93 or 94 or 95 or 96 or 97 or 98 or 99 or 100 or 101 or 102 or 103

105. exp suicidal ideation/ or exp suicide/ or exp suicide attempt/

106. exp automutilation/

107. exp drug overdose/ or exp self poisoning/

108. exp self immolation/

109. (suicid* or "self mutilat*" or "self-mutilat*" or selfmutilat* or "self harm" or "self-harm" or selfharm or "self injur*" or "self-injur*" or selfinjur* or "overdos*" or "self inflict*" or self-inflict* or selfinflict* or "auto mutilat*" or automutilat* or automutilat* or selfcutt* or self-cutt* or "self cutt*" or "head bang*" or headbang* or head-bang* or "self destruct*" or "selfdestruct*" or "self-destruct*" or "self immolat*" or self-immolat* or selfimmolat* or "self poison*" or self-poison* or selfpoison* or parasuicid*).mp. [mp=abstract, heading words, title]

110. 105 or 106 or 107 or 108 or 109

111. 5 and 104 and 110

## **PsycInfo**

1. exp epidemics/

2. exp pandemics/

3. (epidemic or pandemic or outbreak or plague).mp. [mp=abstract, heading words, title]

4. 1 or 2 or 3

5. exp infectious disorders/ or exp bacterial disorders/ or exp parasitic disorders/ or exp viral disorders/

6. exp Hyperthermia/

7. exp Microorganisms/

8. exp Protozoa/

9. exp Encephalitis/

10. exp Gonorrhea/

11. exp Hepatitis/

12. exp HIV/

13. exp AIDS/

14. exp influenza/

15. exp Malaria/

16. exp Meningitis/

17. exp Measles/

18. exp Tuberculosis/

19. exp Gonorrhea/

20. exp Poliomyelitis/

21. exp Creutzfeldt Jakob Syndrome/

22. exp Tuberculosis/

23. (infectio* or fever or viral or virus or bacteria* or fung* or mycosis or mycoses or parasit* or amoeb* or ameb* or arenavir* or botulism or buffalopox or chagas or chikungunya or cholera* or coronavirus or COVID* or SARS* or "acute respiratory syndrome" or campylobacter or corynebacteri* or dengue or diphtheri* or dracunculiasis or "guinea worm" or dysenter* or ebola or "e. coli" or escherichia or elizabethkingia or encephaliti* or enterovir* or flavivirus or francisella or gonococc* or gonorrh* or hantavir* or "hand foot and mouth" or HFMD or orthohantavir* or hepatitis or HIV or AIDS or "human immunodeficiency virus" or "acquired immune deficiency syndrome" or influenza or flu or H1N1 or H1N2 or H2N2 or H3N2 or H3N8 or H5N1 or H7N2 or H7N3 or H7N7 or H9N2 or H10N7 or lassa or leishmania* or leprosy or leptospir* or Weil or legionn* or listeri* or malaria or plasmodium or Marburg or meningiti* or meningococc* or mumps or mycobacteri* or Neisseria or measles or monkeypox or morbilli or MERS or MERS-CoV or "Middle East respiratory syndrome" or myocarditi* or "o'nyong'nyong" or oropouche or pertussis or plague or bubonic or phlebovirus or pneumonic or pseudomonas or aeruginosa or polio* or "rabbit fever" or rabies or rickettsia or "rift valley fever" or salmonell* or "spongiform encephalopathy" or BSE or "mad cow disease" or Creutzfeld-Jakob or "vCJD" or "scarlet fever" or "Seoul orthohantavirus" or "Seoul virus" or SEOV or shigell* or smallpox or streptococc* or staphylococc* or trypanosom* or "sleeping sickness" or toxoplasm* or tuberculo* or TB or tularaemia or tularemia or typhoid or typhus or vibrio or "west nile" or "whooping cough" or yersinia or zika).mp. [mp=abstract, heading words, title]

24. 5 or 6 or 7 or 8 or 9 or 10 or 11 or 12 or 13 or 14 or 15 or 16 or 17 or 18 or 19 or 20 or 21 or 22 or 23

25. exp suicide/ or exp self-destructive behavior/ or exp attempted suicide/ or exp suicidality/ or exp self-injurious behavior/ or exp suicidal ideation/

26. exp Self-Mutilation/

27. exp Self-Destructive Behavior/ or exp Self-Inflicted Wounds/

28. exp drug overdoses/

29. exp Head Banging/

30. exp Self-Poisoning/

31. (suicid* or "self mutilat*" or "self-mutilat*" or selfmutilat* or "self harm" or "self-harm" or selfharm or "self injur*" or "self-injur*" or selfinjur* or "overdos*" or "self inflict*" or self-inflict* or selfinflict* or "auto mutilat*" or automutilat* or automutilat* or selfcutt* or self-cutt* or "self cutt*" or "head bang*" or headbang* or head-bang* or "self destruct*" or "selfdestruct*" or "self-destruct*" or "self immolat*" or self-immolat* or selfimmolat* or "self poison*" or self-poison* or selfpoison* or parasuicid*).mp. [mp=abstract, heading words, title]

32. 25 or 26 or 27 or 28 or 29 or 30 or 31

33. 4 and 24 and 32

## **AMED**

1. exp Disease outbreaks/

2. (epidemic or pandemic or outbreak or plague).mp. [mp=abstract, heading words, title]

3. 1 or 2

4. exp Infection/

5. exp Bacterial infections/

6. exp Fever/

7. exp Viruses/

8. exp Virus disease/

9. exp Bacteria/

10. exp Fungi/

11. exp Mycoses/

12. exp Parasitic disease/

13. exp Amebiasis/

14. exp Cholera/

15. exp Severe acute respiratory syndrome/

16. exp Dysentery/

17. exp Encephalitis/

18. exp hepatitis/

19. exp Hiv/

20. exp Acquired immunodeficiency syndrome/

21. exp Influenza/

22. exp Leprosy/

23. exp Malaria/

24. exp Meningitis/

25. exp Paramyxovirus infections/

26. exp Tuberculosis/

27. exp Measles/

28. exp Whooping cough/

29. exp Poliomyelitis/

30. exp Streptococcal infections/

31. exp Trypanosomiasis/

32. exp Protozoan infections/

33. exp Whooping cough/

34. (infectio* or fever or viral or virus or bacteria* or fung* or mycosis or mycoses or parasit* or amoeb* or ameb* or arenavir* or botulism or buffalopox or chagas or chikungunya or cholera* or coronavirus or COVID* or SARS* or "acute respiratory syndrome" or campylobacter or corynebacteri* or dengue or diphtheri* or dracunculiasis or "guinea worm" or dysenter* or ebola or "e. coli" or escherichia or elizabethkingia or encephaliti* or enterovir* or flavivirus or francisella or gonococc* or gonorrh* or hantavir* or "hand foot and mouth" or HFMD or orthohantavir* or hepatitis or HIV or AIDS or "human immunodeficiency virus" or "acquired immune deficiency syndrome" or influenza or flu or H1N1 or H1N2 or H2N2 or H3N2 or H3N8 or H5N1 or H7N2 or H7N3 or H7N7 or H9N2 or H10N7 or lassa or leishmania* or leprosy or leptospir* or Weil or legionn* or listeri* or malaria or plasmodium or Marburg or meningiti* or meningococc* or mumps or mycobacteri* or Neisseria or measles or monkeypox or morbilli or MERS or MERS-CoV or "Middle East respiratory syndrome" or myocarditi* or "o'nyong'nyong" or oropouche or pertussis or plague or bubonic or phlebovirus or pneumonic or pseudomonas or aeruginosa or polio* or "rabbit fever" or rabies or rickettsia or "rift valley fever" or salmonell* or "spongiform encephalopathy" or BSE or "mad cow disease" or Creutzfeld-Jakob or "vCJD" or "scarlet fever" or "Seoul orthohantavirus" or "Seoul virus" or SEOV or shigell* or smallpox or streptococc* or staphylococc* or trypanosom* or "sleeping sickness" or toxoplasm* or tuberculo* or TB or tularaemia or tularemia or typhoid or typhus or vibrio or "west nile" or "whooping cough" or yersinia or zika).mp. [mp=abstract, heading words, title]

35. 4 or 5 or 6 or 7 or 8 or 9 or 10 or 11 or 12 or 13 or 14 or 15 or 16 or 17 or 18 or 19 or 20 or 21 or 22 or 23 or 24 or 25 or 26 or 27 or 28 or 29 or 30 or 31 or 32 or 33 or 34

36. exp suicide/ or exp suicide attempted/

37. exp Self mutilation/

38. exp Self injurious behavior/

39. 36 or 37 or 38

40. 3 and 35 and 39

**eFigure 1:** Leave-one-out sensitivity analysis for meta-analysis of thoughts of suicide or self-harm (all studies)

**eFigure 2:** Leave-one-out sensitivity analysis for meta-analysis of thoughts of suicide or self-harm (general population)

**eFigure 3:** Leave-one-out sensitivity analysis for meta-analysis of thoughts of suicide or self-harm (healthcare workers)

**eFigure 4:** Leave-one-out sensitivity analysis for meta-analysis of thoughts of suicide or self-harm (psychiatric patients)

**eFigure 5:** Leave-one-out sensitivity analysis for meta-analysis of thoughts of suicide or self-harm (recovered patients)

**eFigure 6:** Sensitivity analysis by study quality for meta-analysis of thoughts of suicide or self-harm
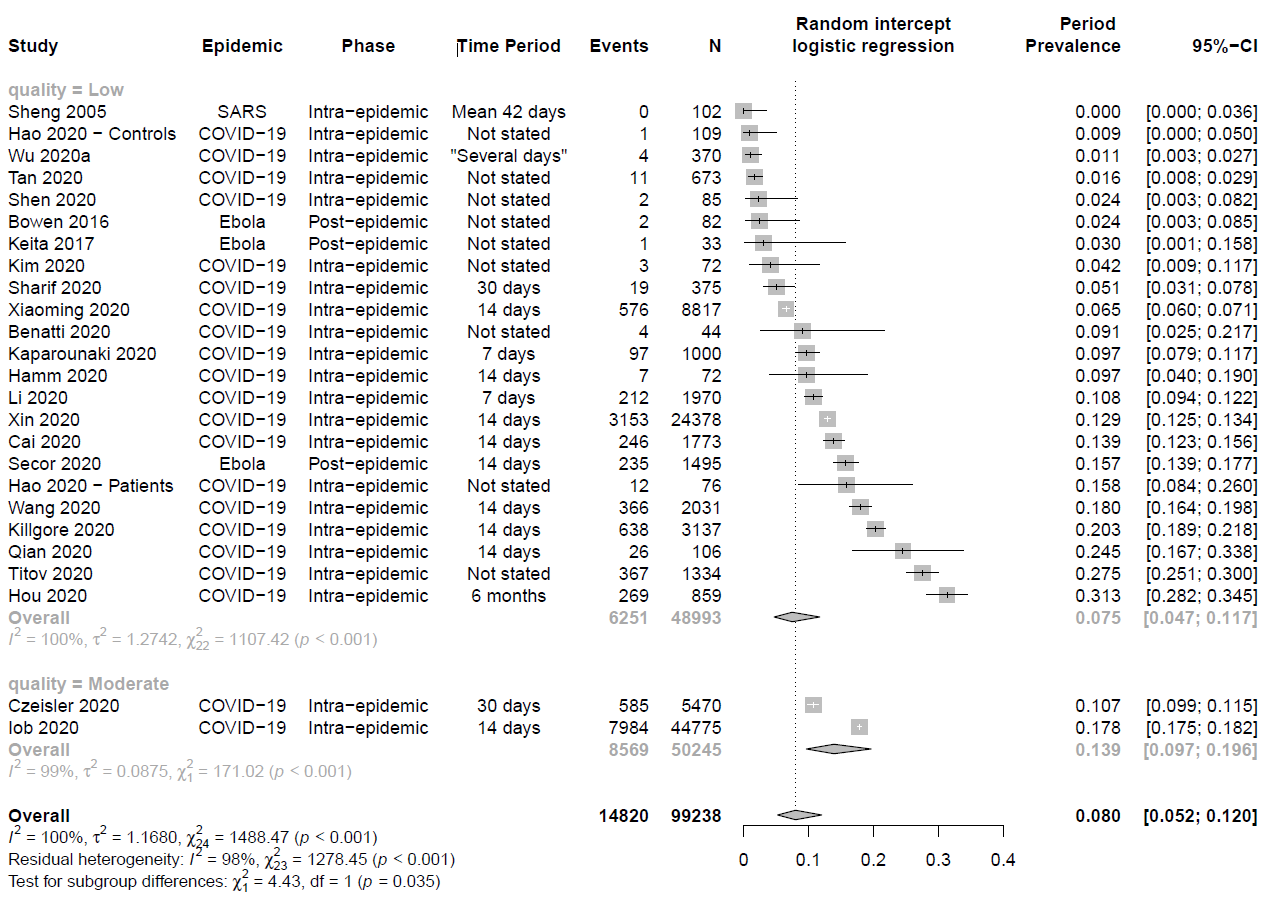


**eFigure 7:** Subgroup analysis of period prevalence for meta-analysis of thoughts of suicide or self-harm
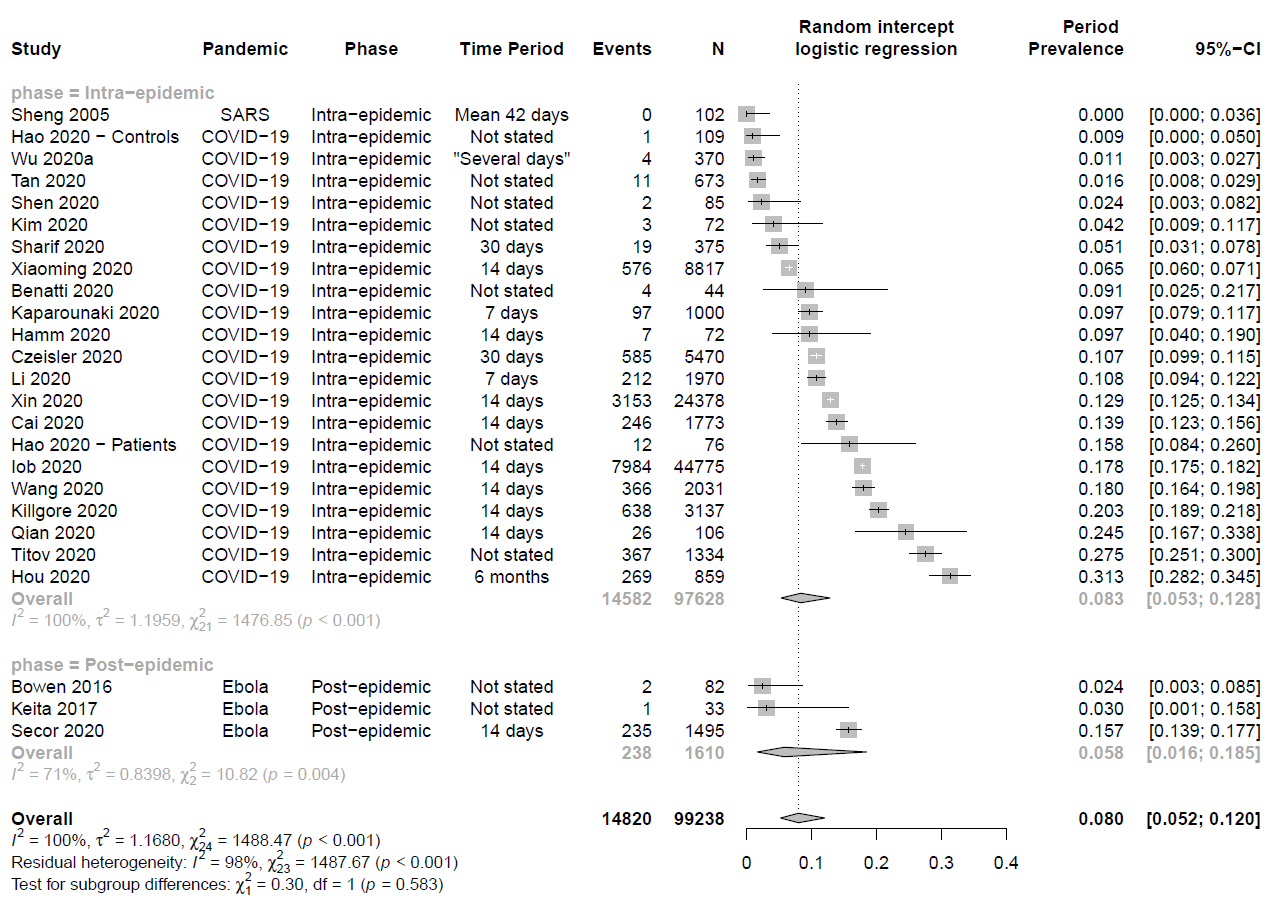

Supplement: Supplementary file 1 [file S2045796021000214sup001.docx]
